# Volatile Composition and Classification of *Paeonia lactiflora* Flower Aroma Types and Identification of the Fragrance-Related Genes

**DOI:** 10.3390/ijms24119410

**Published:** 2023-05-28

**Authors:** Qian Zhao, Lina Gu, Yuqing Li, Hui Zhi, Jianrang Luo, Yanlong Zhang

**Affiliations:** 1 College of Landscape Architecture and Arts, Northwest A&F University, Yangling 712100, China; 2 National Engineering Research Center for Oil Peony, Yangling 712100, China

**Keywords:** herbaceous peony, floral fragrance, volatiles, monoterpenes, phenylethyl alcohol

## Abstract

Flower scent is one of the main ornamental characteristics of herbaceous peony, and the improvement of flower fragrance is a vital objective of herbaceous peony breeding. In this study, 87 herbaceous peony cultivars were divided into three groups (no/light fragrance, medium fragrance, and strong fragrance) based on their sensory evaluation scores, and 16 strong fragrance cultivars and one no fragrance cultivar were selected for subsequent analysis. Sixty-eight volatile components were detected in these 17 cultivars based on solid-phase microextraction (SPME) and gas chromatography/mass spectrometry (GC/MS), and 26 types were identified as important scent components. They were composed of terpenoids, benzenoids/phenylpropanoids, and fatty acid derivatives. According to the content and odor threshold of these main aroma components, the characteristic aroma substances of herbaceous peony were identified, including linalool, geraniol, citronellol, and phenylethyl alcohol (2-PE). The cultivars of strong scented herbaceous peony were divided into three types: rose scent, lily scent, and mixed scent. We explored the possible key genes of characteristic aroma substances in herbaceous peony petals with different odors through the qRT-PCR. The key genes encoding monoterpene biosynthesis were found to be *PlDXS2*, *PlDXR1*, *PlMDS1*, *PlHDR1*, *PlGPPS3,* and *PlGPPS4*. In addition, the linalool synthase (LIS) gene and the geraniol synthase (GES) gene were also found. *PlAADC1*, *PlPAR1,* and *PlMAO1,* related to the biosynthesis of 2-PE were detected, and the synthetic pathway of 2-PE was speculated. In conclusion, these findings revealed that the difference in gene expression of monoterpene and 2-PE synthesis pathway was related to the difference in the fragrance of herbaceous peony. This study explored the releasing pathway of herbaceous peony characteristic aroma substances and provided key genetic resources for fragrance improvement.

## 1. Introduction

Fragrance is an important feature of ornamental plants, which can provide people with a sense of pleasure. Essential oils and spices extracted from aromatic flowers, that can be used in perfume and cosmetics, also increase the economic value of ornamental species [1]. Therefore, it is especially important to pay attention to and strengthen the research on plant flower fragrance. However, since floral fragrance is a complex mixture of small volatile molecules, it is difficult to be evaluated objectively and qualitatively [2]. The collection of volatile compounds is an important step for subsequent analysis and identification, and the commonly used methods include liquid-liquid extraction (LLE), dynamic headspace sampling (DHS), simultaneous distillation extraction (SDE), solid-phase extraction (SPE), and solid-phase microextraction (SPME) [3]. Among them, SPME is a sample pretreatment technology that has developed rapidly in recent years. It has the advantages of solvent-free extraction, fast, efficient, and high sensitivity, and it has been widely used for the analysis of volatile compounds [4].

As far as we know, the floral volatiles identified in horticultural plants are mainly composed of terpenoids, phenylpropanoids/benzenoids, and fatty acid derivatives [5,6]. As the largest group of volatile compounds, terpenes are derived from isopentenyl diphosphate (IPP) and dimethylallyl diphosphate (DMAPP), which are two common and interchangeable precursors [7,8]. Studies have shown that the two precursors are synthesized by the plastid 2-C-methyl-D-erythritol-4-phosphate (MEP) pathway and the cytosolic mevalonate (MVA) pathway [9].

Monoterpenes, the most important volatile terpenes, are synthesized mainly through the MEP pathway in the plastid. The MEP pathway in plants begins with the formation of 1-deoxy-D-xylulose 5-phosphate (DXP) through DXP synthase (DXS) from pyruvate and glyceraldehyde-3-phosphate [8]. At present, the important intermediates in the MEP pathway have been known, and the enzymes that catalyze the conversion of the intermediates have also been successfully identified (as it will argued in the Section 3). Genes involved in the MEP pathway have been the subject of a large number of studies in recent years [8]. The *Arabidopsis* DXS gene can significantly promote the content of monoterpenes in transgenic spike lavender oil [10]. Many terpene synthase (TPS) genes responsible for the formation of volatile terpenes have been identified from many plants, such as *Antirrhinum majus* [11], *Catharanthus roseus* [12], *Clarkia breweri* [13], *Cymbidium* [14], *Freesia* × *hybrid* [15,16], *Lilium* [17], *Osmanthus fragrans* [18], and *Prunus mume* [19]. 

Phenylpropanoids/benzenoids are the second largest class of plant volatile compounds, which originate from 3-phenyl-L-alanine (L-Phe) and are produced by the shikimate and arogenate pathways in plastids [20]. Phenylpropanoids/benzenoids are the main components of flower fragrance in many plant varieties and are also important factors to form a unique scent of flowers [20,21,22]. Phenylethyl alcohol (2-PE) is one of the styrene-phenylpropanoids/benzenoids with pleasant unique rose scent, which is widely used in the production of edible essence [23]. Its metabolic mechanism has also been reported in petunia [24,25], tomato [23,26], and rose [27]. There are three possible biosynthetic pathways of 2-PE [26,27,28], and they will be showed in the “Discussion” section. After L-Phe is converted into phenylacetaldehyde (PALd), PALd is finally converted to 2-PE under the action of alcohol dehydrogenase (ADH) or PALd reductase (PAR). At present, the research on the mechanism of 2-PE synthesis in plants is limited, and there are few reports on some key genes in the synthesis process. More attention will be paid to the 2-PE synthesis pathway and the regulation of 2-PE synthesis at the molecular level in the future [28].

In the vast majority of cases, flower fragrance is a mixture of many compounds, but there are always some main compounds that contribute most significantly to typical scents. Monoterpenes are the characteristic aromatic compounds in *Lilium* ‘Siberia’ [29], benzyl acetate in *Prunus mume* [30], benzenoids in carnation [31], and PALd in petunia ‘TX-794’ [32]. Odor intensity varies greatly. Plants can be classified into groups based on different scents even within a species [33]. Rose is the first flower classified according to its odor compounds, which has led to the fragrance breeding of modern roses and promoted the attraction of rose fragrance in the flower market [34]. Herbaceous peony is a famous traditional Chinese flower, which has a cultivation history exceeding 1500 years [35]. It displays graceful petals and abundant colors, but few cultivars can release pleasant fragrance. Although basic information on herbaceous peony volatile compounds has been obtained in recent years [36,37,38,39], little is known about the classification of aroma types of scented herbaceous peony and the main aroma contributions in herbaceous peony. In addition, there is no research on the emission pattern and synthesis pathway of main aroma contributors. Such information will help to understand the fragrance characteristics of herbaceous peony and can be used for the selection and breeding of new cultivars.

In this study, sensory evaluation was conducted on 87 herbaceous peony cultivars, and 16 cultivars possessed a strong scent were obtained. SPME-gas chromatography/mass spectrometry (GC/MS) was used to identify the volatile components of strong-scented varieties. Sixteen strong-scented varieties were divided into three patterns: a rose scent, a lily scent, and a mixed scent. The key aroma-active compounds were identified by Odor Activity Value (OAV), including linalool, geraniol, citronellol, and 2-PE. In addition, we detected the key genes related to monoterpene and 2-PE biosynthesis and speculated on the metabolic pathway of the main aroma contributing components of herbaceous peony. So far, there have been no reports on the main contributing substances and their formation mechanisms of herbaceous peony fragrance. This study is the first to identify the important contributors of herbaceous peony fragrance by analyzing the volatile compounds, and for the first time, classifying the aroma types of strong-scented herbaceous peony cultivars based on the OAV of the contributing substances. It also explores the pathways and related genes of herbaceous peony aroma synthesis. These results will help us to understand the mechanism of flower fragrance differences among different herbaceous peony cultivars.

## 2. Results

### 2.1. Three Levels of Sensory Evaluation

Sensory evaluation was conducted on eighty-seven cultivars, and seventy-five, eight, and four cultivars belong to the Lactiflora peony cultivar group, Itoh peony cultivar group and Hybrid peony cultivar group, respectively. As shown in Table S1, a total of 16 cultivars with strong fragrance were found, which accounted for 18.39% of all cultivars, of which 15 cultivars belong to the Lactiflora group, and the other one belongs to the Itoh group. There were 27 cultivars (twenty-three Lactiflora peony cultivars and four Itoh peony cultivars) with medium fragrance. Most cultivars had no/light fragrance, accounting for 50.57% of all cultivars. Among them, thirty-seven belong to the Lactiflora group, three belong to the Itoh group, and four belong to the Hybrid group. 

**Figure 1:**
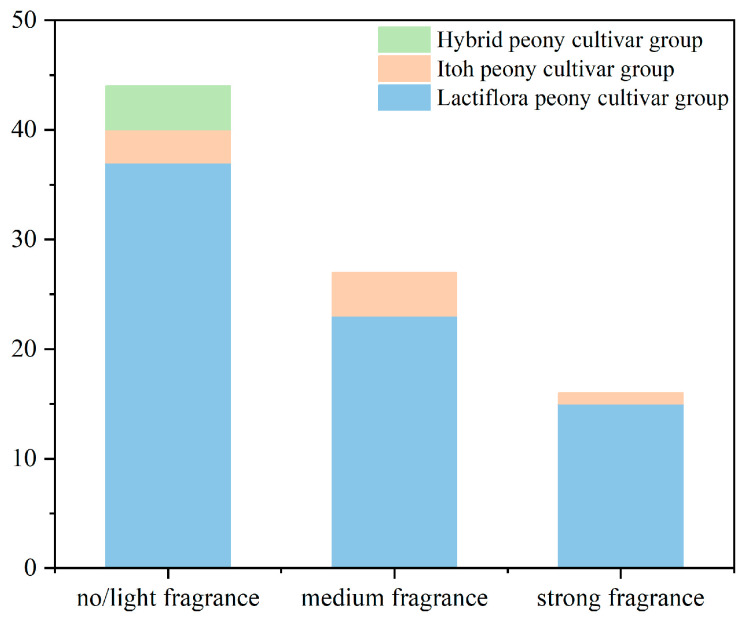
Aroma levels of floral scents in the flowers of 87 herbaceous peony cultivars. The detailed data are shown in the Supplementary Table S1.

The 75 Lactiflora peony cultivars include 15 ones with intense fragrance, 23 ones with medium fragrance, and 37 ones with no/light fragrance. The eight cultivars from the Itoh group include one of intense fragrance, four of medium fragrance, and three of no/light fragrance. All four hybrid peony cultivars had no/light fragrance (Figure 1). Among all these cultivars, ‘Joker’ has the lowest sensory evaluation score and the lowest variance (everyone agreed that this flower does not emit any fragrance).

**Figure 2:**
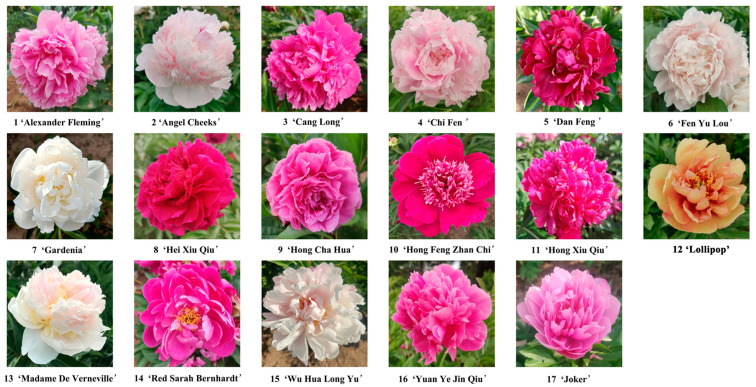
Seventeen herbaceous peony cultivars, which were used for identifying and analyzing the aromatic compounds.

Herbaceous peony with the distinguished aroma was relatively small in these three cultivar groups, and the breeding of aromatic herbaceous peony is indispensable. In order to better understand the differences of volatile compounds in intense fragrance grades and distinguish their odors, all 16 strong fragrance ones were used for subsequent SPEM-GC/MS detection, and ‘Joker’ with no fragrance was used as the control (Figure 2).

### 2.2. Types of Volatile Components from Herbaceous Peony Cultivars

A total of 68 compounds were identified in the 17 herbaceous peony varieties (Table S2). The main volatile components were terpenoids (41), benzenoids/phenylpropanoids (11), and fatty acid derivatives (16). Terpenoids represented the largest proportion of these components (60.94%), followed by fatty acid derivatives (24.57%) and benzenoids/phenylpropanoids (14.49%). Thirty-two types of compounds were newly found in herbaceous peony compared with previous studies [36,37,38,39], and most new compounds were terpenoids (Table S3).

A total of 66 types of volatile compounds were detected in 15 Lactiflora peony cultivars, accounting for 97.14% of all compounds, while 27 types were detected in one Itoh peony cultivar, accounting for 41.43% of all compounds. The only strong aromatic cultivar ‘Lollipop’ of Itoh group is mainly composed of linalool (1968.19 ng/g) and 2-PE (774.73 ng/g). The monoterpene α-Pellandrene and sesquiterpene elemol are the unique components of ‘Lollipop’, among which elemol is firstly identified in herbaceous peony.

**Figure 3:**
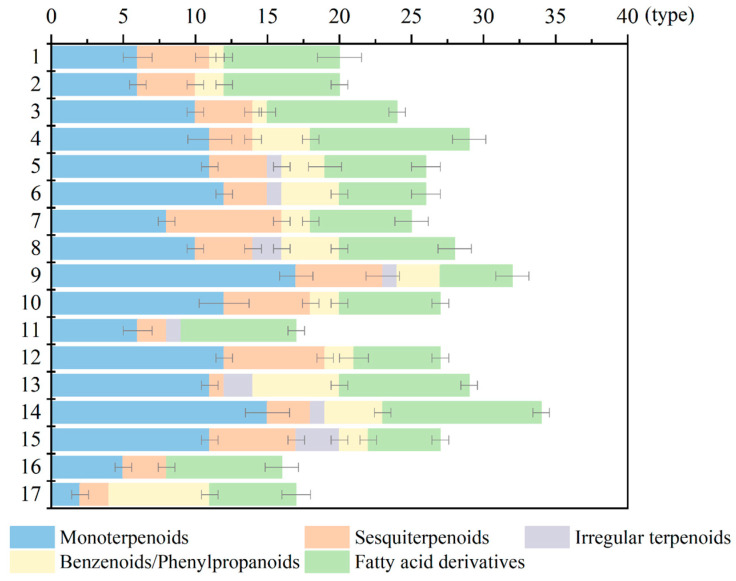
The types of volatile compounds in 17 cultivars. The numbers 1−17 represent 17 cultivars, and numbers are assigned, as described in Figure 2. Three biological replicates of the experiments were carried out. Retain only substances detected in all three biological replicates. The detailed data were shown in Supplementary Table S2.

Obvious differences were found in the types of volatile compounds among different herbaceous peony cultivars (Figure 3). ‘Red Sarah Bernhardt’ and ‘Hong Cha Hua’ contained the most volatile components, 34 and 32 types, respectively. ‘Hong Xiu Qiu’, ‘Yuan Ye Jin Qiu’, and ‘Joker’ contained the least volatile components and only 16–17 types. Each cultivar contained terpenoids, benzenoids/phenylpropanoids, and fatty acid derivatives.

### 2.3. Analysis of Major Volatile Components from Herbaceous Peony Cultivars

Only 26 compounds with relative content greater than 100 ng/g were analyzed further. These compounds accounted for 93.84% of the total volatile compounds, which demonstrated that they were the most important components, including fifteen terpenoids, five benzenoids/phenylpropanoids, and six fatty acid derivatives (Table S4). Monoterpenoids had the highest content among the detected terpenoids.

**Figure 4:**
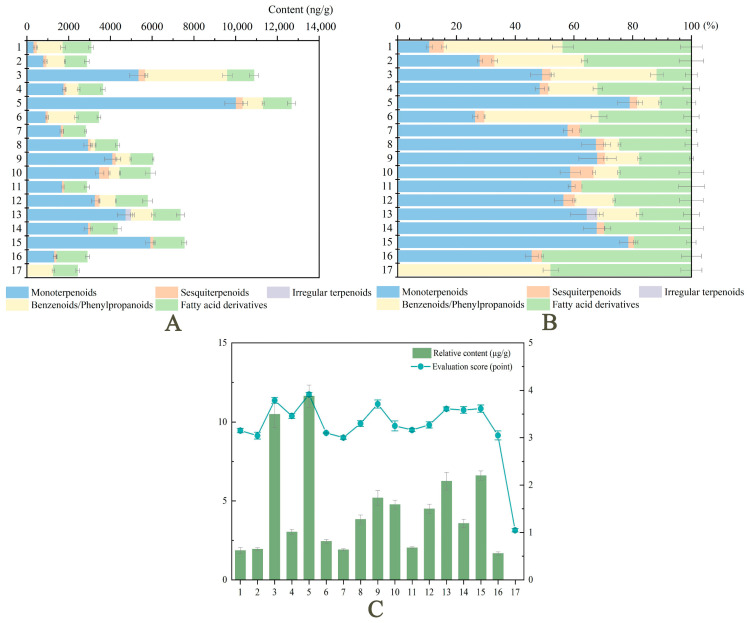
Main volatile compounds of floral fragrance released by 17 herbaceous peony cultivars. 1−17 represents 17 cultivars, and numbers assigned as described in Figure 2. (**A**) The relative contents of 26 main volatile components from 17 cultivars. (**B**) The proportion of 26 main volatile components from 17 cultivars. (**C**) Trend comparison of relative content and sensory evaluation score. Data represent means and standard errors of three biological replicates, and the relative content is calculated using the internal standard method. Detailed data were shown in the Supplementary Table S4.

‘Dan Feng’, ‘Cang Long’, and ‘Wu Hua Long Yu’ possessed the highest contents of volatile components, 12,675.57 ng/g, 10,880.02 ng/g, and 7540.77 ng/g, respectively, which was in agreement with their heavy olfactory fragrance (Figure 4A). The volatile compounds detected in ‘Yuan Ye Jin Qiu’, ‘Hong Xiu Qiu’, ‘Angel Cheeks’, and ‘Gardenia’ were less than 3000 ng/g. Among the 16 cultivars with strong fragrance, monoterpenoids accounted for the high proportion, except for ‘Alexander Fleming’, ‘Angel Cheeks’, and ‘Fen Yu Lou’, which were dominated by benzenoids/phenylpropanoids and fatty acid derivatives. No terpenoids were detected in the control ‘Joker’ (Figure 4B). Removing the volatile components that released from ‘Joker’, we found that the release of aromatic compounds was basically consistent with the trend of sensory evaluation (Figure 4C). 

The composition of floral scent in each cultivar is particularly different. Twenty-five volatile compounds were considered as the major scent components of 16 strong scented cultivars, nine of which were more than 10% in according cultivars, including six monoterpenoids (α-pinene, linalool, myrtenal, citronellol, citral, and geraniol), one benzenoids/phenylpropanoids (2-PE), and two fatty acid derivatives (hexanal and 2-hexenal) (Table S4). Hexanal and 2-hexenal were the components detected in all 17 cultivars. Geraniol was detected in 15 cultivars. Linalool and citral were found in 13 cultivars. Citronellol and 2-PE were also widely detected in 11 cultivars.

### 2.4. Odor Classification and OAVs of Volatile Compounds

**Figure 5:**
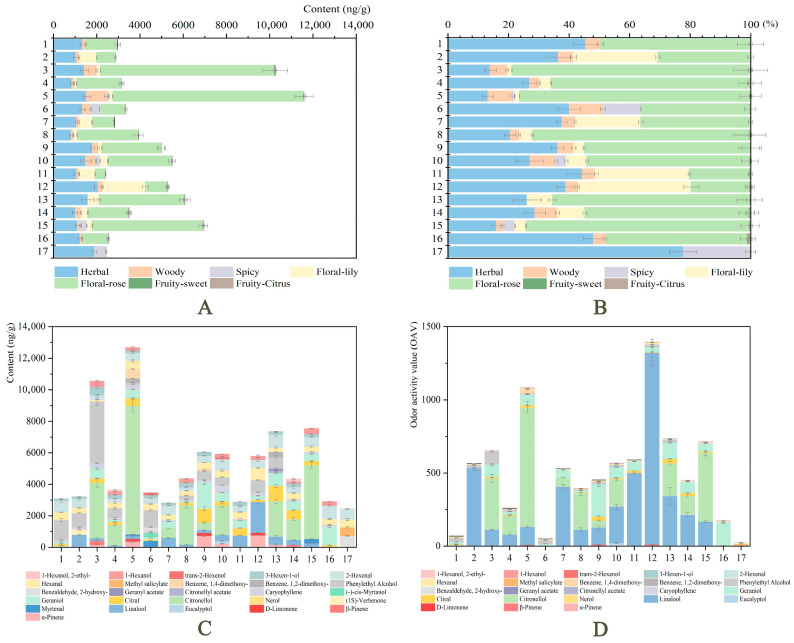
The odor classification, content, and OAV of 26 main volatile compounds from 17 cultivars. The numbers 1−17 represent 17 cultivars, and numbers are assigned, as described in Figure 2. (**A**) The relative contents of main volatile compounds in seven groups. (**B**) The proportion of main volatile components in seven groups. (**C**) The relative contents of 26 main volatile compounds. (**D**) The OAVs of 26 main volatile compounds. Data represent means and standard errors of three biological replicates. The detailed data were shown in Supplementary Tables S4 and S5.

Major volatile compounds were divided into seven groups (herbal, woody, spicy, floral-rose, floral-lily, fruity-sweet, and fruity-citrus) according to their odor descriptions (Table S5). The total content of seven groups and their percentage in the total volatile content of 17 cultivars were shown in Figure 5A,B. Herbal, floral-rose, and floral-lily were the three groups with the highest proportion and contents.

However, there is no direct relationship between the content of volatile compounds and their aroma contribution, and the odor threshold should also be considered. OAV is the ratio of the volatile contents to its odor threshold, which is an important indicator to measure the aroma contribution [40]. The greater the volatile OAVs, the greater the contribution to aroma [41].

Volatile compounds with OAV ≥ 1 were generally considered as effective contributors to overall aroma [41]. As shown in Table S5, the OAV of 17 volatiles was greater than 1, indicating that they were contributors to the aroma of herbaceous peony. Compounds with OAV ≥ 10 were considered as the important aroma substances [41]. Eight compounds were the important components contributing to the aroma of herbaceous peony. Four compounds had very high OAVs (> 80) and may be the most important odor compounds for the overall aroma, including linalool, citronellol, geraniol, and 2-PE.

Combined with the contents of 26 volatile components of 17 cultivars (Figure 5C), some substances, such as fatty acid derivatives, were found with a high odor threshold. Although their content was high, the contribution to aroma is small. In addition, the content of fatty acid derivatives in 17 cultivars was concentrated in 1000–1500 ng/g with no significant difference, and they were distributed in six types (hexanal, 2-hexenal, 3-hexen-1-ol, trans-2-hexenol, 1-hexanol, 1-hexanol, and 2-ethyl-). Most of these types release grass fragrance, which is not considered a component of flower fragrance. The reason for the sensory difference of flower fragrance among herbaceous peony cultivars was mainly due to the different contents of terpenoids and benzenoids/phenylpropanoids.

Linalool and citronellol were the main aroma contribution components of most strong-scented herbaceous peony cultivars, and geraniol and 2-PE were also the main aroma contribution components of a few cultivars based on the OAVs of volatile compounds (Figure 5D).

### 2.5. Grouping of Herbaceous Peony Cultivars Based on Their Fragrance

**Figure 6:**
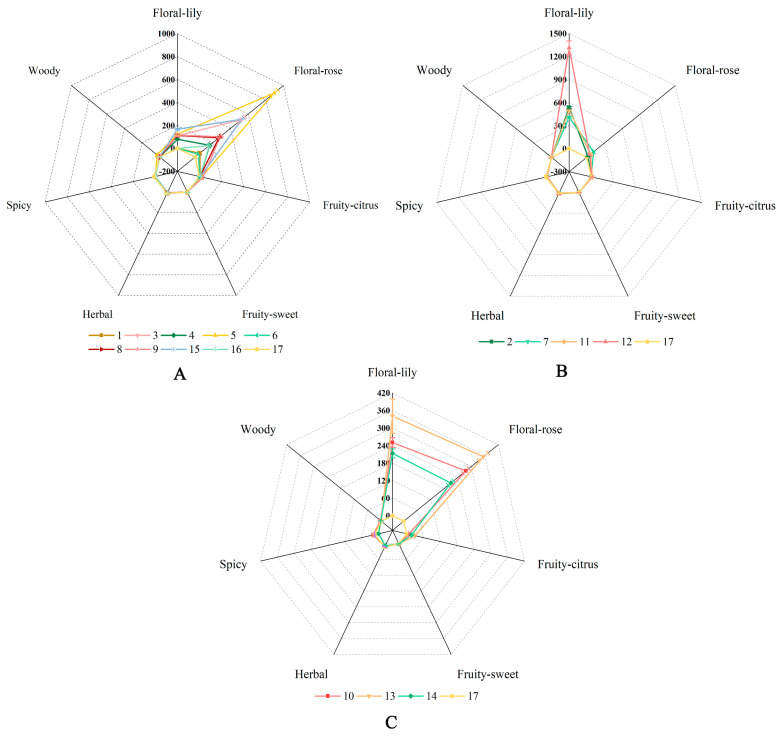
The radar chart of volatile characteristics of 17 herbaceous peony cultivars. The numbers 1−17 represent 17 cultivars, and numbers are assigned, as described in Figure 2. (**A**) A rose scent. (**B**) A lily scent. (**C**) A mixed scent. Data represent means and standard errors of three biological replicates. The detailed data are shown in the Supplementary Table S5.

Citronellol, 2-PE, and geraniol belong to the floral-rose group, while linalool belongs to the floral-lily group, and the scent of varieties were divided into three types according to the OAVs of volatile compounds. The aroma profile was shown in Figure 6 to visually display the aroma characteristics of each type. The OAVs of the aromatic compounds in ‘Joker’ are very low and do not emit any fragrance, which is used as the control.

- A rose scent

Most herbaceous peony cultivars belong to this aroma type, including ‘Alexander Fleming’, ‘Cang Long’, ‘Chi Fen’, ‘Dan Feng’, ‘Fen Yu Lou’, ‘Hei Xiu Qiu’, ‘Hong Cha Hua’, ‘Wu Hua Long Yu’, and ‘Yuan Ye Jin Qiu’ (Figure 6A). Citronellol, 2-PE, and geraniol emitted a rose-like scent [42,43]. These cultivars were described as roselike fragrances, while citronellol, 2-PE, and geraniol were detected as their major aromatic contribution compounds.

- A lily scent

‘Angel Cheeks’, ‘Gardenia’, ‘Hong Xiu Qiu’, and ‘Lollipop’ were grouped into lily scent group (Figure 6B). Linalool was a natural spice with the flavor of lily [17,42]. Linalool was detected as the major aromatic contribution compounds in these cultivars, which was described as a fragrance resembling lily.

- A mixed scent

The contribution rate of floral-rose substances was equal to that of floral-lily substances in ‘Hong Feng Zhan Chi’, ‘Madame De Verneville’, and ‘Red Sarah Bernhardt’ (Figure 6C). These cultivars were described as a mixed scent [17,42,43].

### 2.6. Analysis of Genes Related to Monoterpene Synthesis

**Figure 7:**
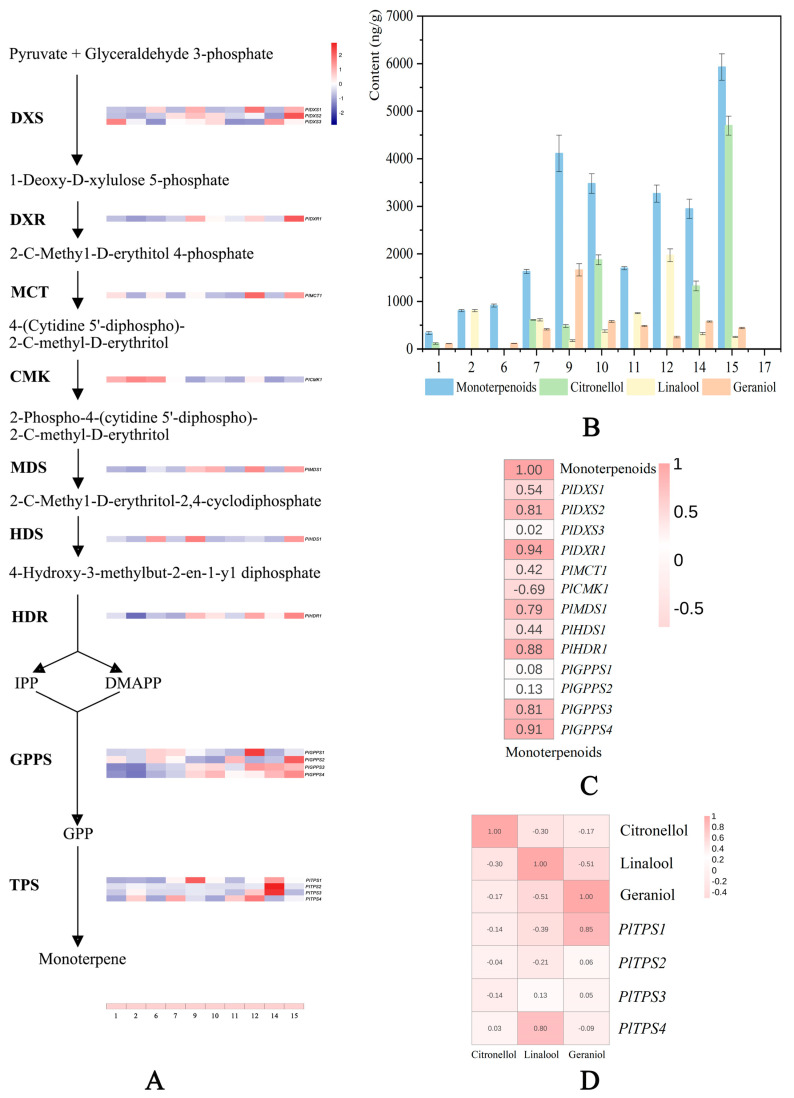
MEP pathway in herbaceous peony. The numbers 1−17 represent 17 cultivars, and numbers assigned are described in Figure 2. (**A**) Expression pattern of genes involved in the MEP pathway of herbaceous peony. Gene expression levels in different cultivars are represented by color gradations. (**B**) The relative contents of monoterpenes, citronellol, linalool, and geraniol in 11 cultivars. The detailed data are sourced from Figure 4 and Figure 5 and Supplementary Table S4. (**C**) Correlation heat map between monoterpenes release and gene expression levels. Correlation levels are represented by color gradations. (**D**) Correlation heat map between citronellol, linalool, and geraniol releases and TPS gene expression levels. Correlation levels are represented by color gradations. Three biological replicates of the experiments were carried out.

Plant metabolism can be divided into primary metabolism, which contains many crucial reactions and pathways, with the main function of maintaining plant survival, and secondary metabolism, which mainly involves the interaction between plants and the environment, achieving many important functions in the growth and development [44]. Some primary metabolites, which are produced through glycolysis, the TCA cycle, or the shikimate pathway, can serve as precursors to synthesize secondary metabolites, such as terpenoids, phenols, and nitrogen/sulfur-containing compounds [45,46]. Terpenoids are the largest family of secondary metabolites, and volatile terpenoids, such as monoterpenes and sesquiterpenes, greatly contribute to the aroma of flowers and several fruits [47]. In plants, there are two pathways involved in terpene synthesis: the MEP pathway and the MVA pathway. Citronellol, geraniol, and linalool were monoterpenes, and they were synthesized by seven enzymatic steps in the MEP pathway [48]. Two primary metabolites, pyruvate and glyceraldehyde-3-phosphate, derived from glycolysis and the pentose phosphate pathway, were the substrates of the first enzyme (DXS) of the MEP pathway [44]. Nine related genes were detected in the upstream of MEP pathway in the transcriptome database of *Paeonia lactiflora* (accession number SRP287892), including three DXSs, one DXR, one MCT, one CMK, one MDS, one HDS, and one HDR (Figure 7A).

RNA from flowers in full bloom of cultivars ‘Alexander Fleming’, ‘Angel Cheeks’, ‘Fen Yu Lou’, ‘Gardenia’, ‘Hong Cha Hua’, ‘Hong Feng Zhan Chi’, ‘Hong Xiu Oiu’, ‘Lollipop’, ‘Red Sarah Bernhardt’, ‘Wu Hua Long Yu’, and ‘Joker’ were extracted. Among them, cultivars ‘Alexander Fleming’, ‘Angel Cheeks’, and ‘Fen Yu Lou’ released slight monoterpenoids (<1000 ng/g), the cultivars ‘Gardenia’, ‘Hong Xiu Oiu’, and ‘Red Sarah Bernhardt’ released medium monoterpenoids (1000 ng/g–3000 ng/g), while cultivars ‘Hong Cha Hua’, ‘Hong Feng Zhan Chi’, ‘Lollipop’, and ‘Wu Hua Long Yu’ released a large amount of monoterpenoids (>3000 ng/g). The cultivar ‘Joker’ did not release any monoterpenes as the control (Figure 7B).

The qRT-PCR analysis of nine monoterpene synthesis-related genes in eleven cultivars were carried out (the expression level of ‘Joker’ was set as 1), and it was finally found that *PlDXS2*, *PlDXR1*, *PlMDS1,* and *PlHDR1* were strongly correlated with the monoterpene release content of these 11 cultivars (Figure 7C). It is speculated that these genes may be the key genes for the monoterpene synthesis of herbaceous peony. 

Other compounds, such as geranyl pyrophosphate (GPP), the precursor of monoterpenoid synthesis, is generated by GPP synthase (GPPS) [49]. Four GPPSs were found in the *Paeonia lactiflora* transcriptome, and the expression level of *PlGPPS3* and *PlGPPS4* was strongly correlated with monoterpene release (Figure 7C). 

Lastly, a series of monoterpenes are synthesized through different TPSs using substrate GPP [48]. Different TPS genes can catalyze the formation of different monoterpenes. Four TPS genes related to monoterpenes synthesis were selected in the transcriptome. The expression of *PlTPS1* was similar to the release law of geraniol in 11 cultivars, while the expression of *PlTPS4* was similar to the release law of linalool (Figure 7D). *PlTPS2* and *PlTPS3* were highly expressed only in ‘Red Sarah Bernhardt’ and may be related to β-pinene produced only in ‘Red Sarah Bernhardt’.

### 2.7. Analysis of Genes Related to 2-PE Synthesis

**Figure 8:**
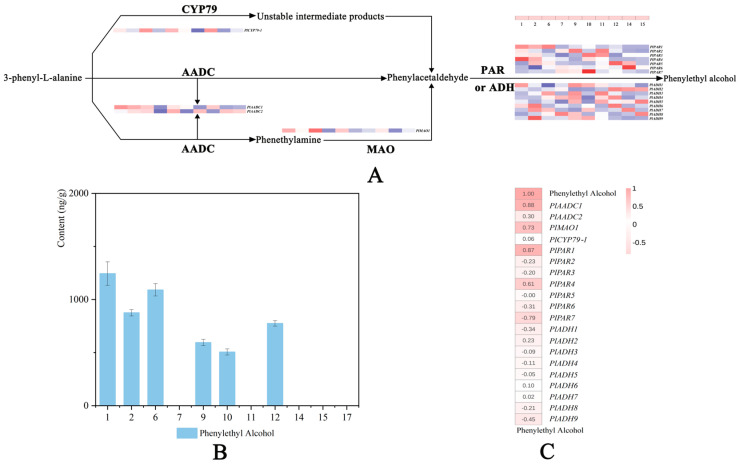
2-PE synthesis pathway in herbaceous peony. The numbers 1−17 represent 17 cultivars, and numbers are assigned, as described in Figure 2. (**A**) Expression pattern of genes involved in the three possible 2-PE synthesis pathways of herbaceous peony. Gene expression levels in different cultivars are represented by color gradations. (**B**) The relative contents of 2-PE in 11 cultivars. The detailed data are sourced from Figure 4 and Figure 5 and Supplementary Table S4. (**C**) Correlation heat map between 2-PE release and gene expression levels. Correlation levels are represented by color gradations. Three biological replicates of the experiments were carried out.

As the main aroma contribution of cultivars ‘Alexander Fleming’ and ‘Fen Yu Lou’, the synthetic pathway of 2-PE in herbaceous peony is also worth analyzing. As we all know, there are generally three pathways for the synthesis of 2-PE [50]. The corresponding genes in three possible biosynthetic pathways were selected, including two AADCs, one MAO, one CYP79, seven PARs, and nine ADHs (Figure 8A). 

Among the cultivars that extract RNA, cultivars ‘Gardenia’, ‘Hong Xiu Oiu’, ‘Red Sarah Bernhardt’, and ‘Wu Hua Long Yu’ did not release 2-PE, the cultivars ‘Angel Cheeks’, ‘Hong Cha Hua’, ‘Hong Feng Zhan Chi’, and ‘Lollipop’ released slight 2-PE (<1000 ng/g), while cultivars ‘Alexander Fleming’ and ‘Fen Yu Lou’ released a large amount of 2-PE (>1000 ng/g). ‘Joker’ was the control (Figure 8B).

The expression pattern of *PlCYP79-1* has no correlation with the release of 2-PE, indicating that the synthesis of 2-PE in herbaceous peony should not follow the first pathway. The expression patterns of *PlAADC1* and *PlMAO1* were strongly correlated with the release of 2-PE. We speculate that the synthesis of 2-PE in herbaceous peony followed the second or third way. *PlAADC1* and *PlMAO1* may play important roles in the conversion of L-Phe to PALd. There was no correlation between *PlADH*s and 2-PE release, while *PlPAR1* and *PlPAR4* were positively correlated with 2-PE release. *PlPAR1* and *PlPAR4* may be the key genes for PALd to synthesize 2-PE (Figure 8C). 

## 3. Discussion

### 3.1. Complexity and Diverse of Herbaceous Peony Floral Aroma Compounds

Based on the sensory evaluation of 87 herbaceous peony cultivars, the small proportion of aromatic ones in the three cultivar groups was found. Only 16 cultivars released strong fragrance, and most of the 87 cultivars had no/light fragrance, accounting for 50.57% of all varieties. The cultivation of aromatic herbaceous peony is indispensable.

The mixture of volatile compounds produces a variety of flower fragrances [48]. Aromatic herbaceous peony also has differences in volatile components to give people different olfactory experiences. The development of sensitive analytical methods, such as SPME, DHS, and GC/MS, has made it easy to collect and identify volatile compounds. These methods have been used to study the aromas of tree peony [3,20,42], grape [51], rose [43,52], and lily [17]. In this study, 68 volatile components of 17 cultivars (16 strong fragrance ones and the Joker with no fragrance as the control) were collected through SPEM and analyzed by GC/MS, of which 32 were reported for the first time. The differences may be caused by the different varieties and detection methods. Terpenes, benzenoids/phenylpropanoids, and fatty acid derivatives were the main chemical categories. These strong-scented varieties could be used as breeding parents to produce aromatic generations and have high economic value as essences, spices, or teas, which can be used in the development of flavor food and other fields using aromas. 

A total of 26 compounds with a relative content more than 100 ng/g (93.84% of the total flower fragrance) were further analyzed. These compounds were considered to be the most important volatile compounds in the composition of herbaceous peony fragrance. Monoterpenes were the main substances released by most cultivars, while a few cultivars, ‘Alexander Fleming’, ‘Angel Cheeks’, and ‘Fen Yu Lou’, were dominated by benzene/phenylpropanoids and fatty acid derivatives (Figure 4A). The order of total volatile release of each cultivar is basically consistent with the results of aroma evaluation, indicating that sensory perception is closely related to volatile compound contents. 

The characteristic aroma components play a determining role in the aroma of plants. The most commonly used method to identify the characteristic aroma components is the flavor threshold method, which is mainly used in the aroma analysis of edible products, and also in plants [53,54,55]. The characteristic aroma components of *chrysanthemum* ‘Jinba’ were isocyclocitral, eucalyptus alcohol, α-pinene, β-farnesene, and caryophyllene [56]. Cineole, β-myrcene, and β-ocimene were the characteristic aroma components of *Rhododendron fortune* [57]. Β-Myrcene, D-limonene, β-ocimene, linalool, and 2-PE were the characteristic aromas of Begonia [55]. The greater the OAV, the higher the contribution of this compound to aroma. In this study, seventeen characteristic aroma components were identified, among which four have very high OAVs (>80) and were recognized as the important characteristic aroma components of herbaceous peony flower fragrance, including linalool, citronellol, geraniol, and 2-PE (Table S5). Fatty acid derivatives were not considered as characteristic substances due to the low OAVs and the small difference in their total content among different sensory cultivars. The diversity of herbaceous peony flower fragrance is mainly due to the different release of monoterpenes and benzene/phenylpropanoids.

Although monoterpenes and benzene/phenylpropanoids have been reported to be the main compounds released from herbaceous peony [36,37,38,39], the characteristic aroma substances are still unclear. This study identified the main contribution substances and analyzed the main reasons for the sensory differences of herbaceous peony flower fragrance and found that a large amount of released fatty acid derivatives were not the main contribution volatiles. 

The classification of aroma types is challenging, and it is hard for the human nose to describe the odor characteristics of flowers. The level of flower fragrance is simply described as non-scented, light scented, and strongly scented [17]. The classification of rose fragrance types promotes the breeding of rose fragrance [34]. In other ornamental plants, flower fragrance has been similarly classified to encourage further efforts to breed fragrant plants, such as Dianthus [58], tulip [33], and lily [17]. In our study, volatile compounds were grouped into seven categories based on the odor description. Based on the composition and contribution of main aroma components, referring to the classification methods of lily and tulip [17,33], 16 cultivars were grouped into three categories: rose scent, lily scent, and mixed scent. As far as we know, this is the first time to classify herbaceous peony cultivars with strong scent based on the aroma compounds contribution, which also provides a reference for the subsequent improvement of herbaceous peony flower fragrance and the cultivation of new cultivars with rose or lily scent.

### 3.2. Terpene Biosynthesis-Related Genes Contribute to the Fragrance of Herbaceous Peony

The research of herbaceous peony flower fragrance mainly focused on the recognition of volatile components, but the molecular mechanism of its formation is still unclear. Monoterpenes and 2-PE play leading roles in the fragrance of herbaceous peony. Their different emission patterns may make the most significant contribution to the flavor difference between different cultivars. Thus, exploring the expression level of different genes in the monoterpene and 2-PE biosynthetic pathways is crucial to illuminate the key genes of flower scent metabolism. The MEP pathway is the major monoterpene synthesis pathway in plants, and the genes that play a pivotal role in MEP pathway regulate the synthesis of monoterpenoids [9]. Therefore, studying these key genes is of great importance to reveal the molecular mechanism of monoterpene biosynthesis. Genes related to the MEP pathway were found from the transcriptome database. By comparing the gene expression of strong scented herbaceous peony cultivars with that of non-scented cultivar, *PlDXS2*, *PlDXR1*, *PlHDS1*, *PlHDR1*, *PlGPPS3,* and *PlGPPS4* in strong scented cultivars with monoterpene release were up-regulated compared with the control (Figure 7A).

DXS is a rate-limiting enzyme in the MEP pathway, which catalyzes the first step and controls the synthesis of terpenes. Huang et al. [15] found that *FhDXS2A* played an important role in terpene biosynthesis. *HcDXS2A* of *Hedychium coronarium* was consistent with the release of monoterpenoids [59]. The expression level of *PlDXS2* was higher in the cultivars with large release of monoterpenes. Its expression increased, thus producing sufficient substrates for the downstream DXR to drive MEP pathway. DXR genes of *Mentha piperita* contributed to the biosynthesis of monoterpene compounds [60]. After the overexpression of DXR gene into *Tripterygium wilfordii*, begonin accumulated rapidly [61]. HDS is an enzyme protein that catalyzes the sixth step of the MEP pathway and plays an important role in the synthesis of monoterpenes. The HDS gene is related to the synthesis of aromatic substances in *Narcissus tazetta* [62]. HDS in *Artemisia annua* regulated the synthesis of artemisinin [63]. The *LiHDS* gene participated in the monoterpene synthesis of *Lilium* ‘Siberia’ [64]. HDR catalyzes the synthesis of IPP and DMAPP, provides precursors in the MEP pathway, and plays a role in regulation and speed limitation. The HDR gene of calendula maintained a constant ratio of IPP and DMAPP [65]. *GbHDR* of *Ginkgo biloba* played a key role in the synthesis and accumulation of terpenes [66]. In this study, the expression patterns of *PlDXS2*, *PlDXR1*, *PlHDS1,* and *PlHDR1* genes in the upstream of the MEP pathway were consistent with the monoterpene release trend (Figure 7C).

GPPS mainly provides precursors for the synthesis of monoterpenes [67]. GPPS is involved in monoterpene production in various plant species, including *Arabidopsis* [68], *Clarkia breweri* [69], *Humulus lupulus* [70], and *Phalaenopsis bellina* [71]. In the downstream MEP pathway, two GPPS gene expression levels were consistent with the monoterpene release trend in 16 cultivars.

The expression of *PlDXS2*, *PlDXR1*, *PlHDS1*, *PlHDR1*, *PlGPPS3,* and *PlGPPS4* in herbaceous peony cultivars with high monoterpene release was higher than those with low monoterpene release, indicating that the activation of the MEP pathway of high monoterpene released cultivars was high, thus accelerating the accumulation of GPP, the precursor of monoterpene synthesis (Figure 7A).

As the key enzyme of terpene synthesis, TPS can catalyze different substrates to produce different products [20]. GPP can form monoterpenes under the catalysis of TPS [11]. TPS genes have been identified in many plant species. *FhTPS1* is a mono-product enzyme that catalyzes the formation of linalool [16]. Both *LoTPS1* and *LoTPS3* catalyze the formation of linalool, and the former can also specifically catalyze the formation of (Z)-β-ocimene, and the latter is a promiscuous monoterpene synthase that can also catalyze the formation of cis-nerolidol [72]. In our study, the expression pattern of *PlTPS1* is similar to release amount of geraniol, while the expression pattern of *PlTPS4* is similar to that of linalool (Figure 7D). It is speculated that *PlTPS1* is a geraniol synthase (GES) gene, and *PlTPS4* is a linalool synthase (LIS) gene. The TPS gene related to citronellol synthesis has not been found, and the synthesis of citronellol in herbaceous peony remains to be studied. 

### 3.3. 2-PE Biosynthesis-Related Genes Contribute to the Fragrance of Herbaceous Peony

2-PE was first discovered as a characteristic aroma compound in plant flowers. It is a simple aromatic primary alcohol with elegant, delicate, and lasting rose fragrance [50]. The synthesis of 2-PE, including three pathways. Tieman [26] found that the synthesis of 2-PE in tomato followed the second way. The key enzymes were AADC, MAO, and PAR [23,26]. The synthesis of 2-PE in rose followed the third way petunia [27]. *RrPAR* and *RrAADC* genes of rose can promote the synthesis and accumulation of 2-PE [43]. AADC in grape can promote the synthesis of 2-PE from L-PHe [50]. Twenty genes in the transcriptome database that may be related to 2-PE synthesis were screened for subsequent qRT-PCR analysis. *PlAADC1*, *PlPAR1*, *PlPAR4,* and *PlMAO1* were found to have a strong correlation with the release trend of 2-PE (Figure 8C). By characterizing the related genes in the three possible pathways of 2-PE synthesis, it is speculated that the synthesis of 2-PE in herbaceous peony basically conforms to the second or third pathway. PAR is the key gene for PALd to synthesize 2-PE. Although there have been many studies on plant volatile components in recent years, there are few reports on 2-PE, especially on the regulation of 2-PE synthesis in plants. This study speculated the synthesis pathway of 2-PE in herbaceous peony at the molecular level, providing a reference for the future breeding of aromatic herbaceous peony or improving the content of 2-PE in herbaceous peony. 

## 4. Materials and Methods

### 4.1. Plant Materials

Eighty-seven herbaceous peony (*Paeonia lactiflora* Pall.) cultivars, including Lactiflora peony cultivar group, Hybrid peony cultivar group, and Itoh peony cultivar group, were used in the study (Table S1). Plants were grown in the field of Northwest Agriculture and Forestry University, Yangling, Shanxi, China.

### 4.2. Sensory Evaluation of Herbaceous Peony Fragrance

Flowers of the same size in full bloom were collected in batches from 10:00 a.m. to 11:00 a.m. on sunny days from April 21 to May 11, according to the florescence of herbaceous peony in 2022. The samples were randomly numbered and taken back to the laboratory, and the branches with blooming flowers were inserted into water at room temperature. Twenty-four participants, aged 20–40, were all in good health, as well as free from bad habits and pollen allergy. Participants promised not to smell and use irritating odor substances, not to smear substances with fragrance, and to keep calm before the experiment. Sensory evaluation was conducted on 87 varieties of herbaceous peony.

The fragrance of each sample was scored according to the evaluation criteria of three grades: no/light fragrance (1 < score ≤ 2), medium fragrance (2 < score ≤ 3), and strong fragrance (3 < score ≤ 4). Participants need to rest for 1–2 min after each evaluation of 3 samples. The final score of each variety is determined by the average of all scores. These scores were obtained after excluding abnormal values through Dixon’s Q test. 

### 4.3. Floral Scent Collection and Analysis

Petals were collected at the full-opened stage (the first day the flower bloom from half open to full open). To avoid the closed state of flowers, the samples were collected from 10:00–11:00 in the sunny morning. Floral scent collections were conducted using the headspace SPEM (HS-SPME) method and performed with three replicates. This method has been proven to be linear in the range of 5–200 mg/l, and all R2 values were above 0.99. The minimum limit of detection was less than 4 × 10^−3^ ng. In five repeated experiments, it was found that the standard sample recovery rate was over 80%, and the relative standard deviation of the main volatile components was under 10%, indicating good reproducibility [73,74]. This method can accurately and effectively analyze volatile aromatic substances. 

The SPME fiber (50/30 µm, DVB/CAR/PDMS fiber, Supelco, Bellefonte, PA, USA) was aged for 2 h in the GC injection (250 °C). After cutting the entire fresh herbaceous peony petals, the inner and outer petals were evenly mixed, and then accurately weighed at 0.5 g. Petals were put into a 40mL collection bottle with 20 µL 3-octanal (82 ng/µL) as the internal standard. The bottle was placed on the magnetic stirrer. The aged SPME fiber was inserted into the headspace of the bottle and was extracted at 40 °C for 40 min. Then, the fiber was inserted into the GC/MS (ISQ & TRACE GC Ultra, Thermo Fisher Scientific, Waltham, MA, USA) system and desorbed at 250 °C for 3 min. Three biological replicates of the experiments were carried out. 

GC conditions are as follows. Carrier gas: helium; chromatographic column: DB-5MS (30 m × 0.25 mm × 0.25 µm, Agilent, Palo Alto, CA, USA); carrier gas flow rate: 1 mL/min; injection temperature: 250 °C; injection mode: no split flow; column temperature: 40 °C for 2.5 min, increased from 40 °C to 230 °C at a rate of 5 °C/min, and then held for 5 min. MS conditions are as follows. Electron ionization: EI source, 70 eV; mass range: 35–450 m/z.

The volatile compounds in each sample were identified by the combination of the National Institute of Standards and Technology (NIST) library. The identification results were selected with a Match Factor (SI) or Reverse Match Factor (RSI) thresholds greater than 800 (900 and above, mass spectrometry matching is excellent; 800–900, good; 700–800, fair; <600, Poor), and they were ultimately determined based on the retention index. The content of each component is determined by the internal standard method. The calculation formula is as follows: each component content (ng/g) = [(peak area of each component × internal standard content (ng/µL) ×internal standard volume (µL)/peak area of internal standard]/sample weight (g). 

### 4.4. Classification of Scents

The odor description of volatile compounds was based on references [17,43,75]. The odor thresholds in water were mainly referred to Van Gemert [76] and online database (http://www.vcf-online.nl/VcfHome.cfm) (accessed on 10 March 2023). OAVs are calculated as follows: OAVs = each component content (ng/g)/odor threshold of volatile compounds (mg/kg). 

### 4.5. RNA Isolation and cDNA Synthesis

The total RNA of petals was extracted following the RNAprep Pure Plant Kit (TIANGEN Biotech Co. Ltd., Beijing, China). The degradation and quality of total RNA were examined through agarose gel electrophoresis and NanoDrop 2000 spectrophotometer (Thermo Fisher Scientific, Waltham, MA, USA). First-strand cDNA was synthesized following the PrimeScript RT Reagent Kit with gDNA Eraser (Takara Bio. Inc., Dalian, China).

### 4.6. Expression Analysis of Related Genes by qRT-PCR

The expression of related genes was detected by qRT-PCR using the SYBR Premix Ex Taq Kit (Takara Bio. Inc., Dalian, China) and performed with three replicates. The expression levels of related genes were calculated by the 2^−ΔΔCT^ method and *PlGADPH* as a reference. The specific primers of selected genes were listed in Table S6. 

## 5. Conclusions

**Figure 9:**
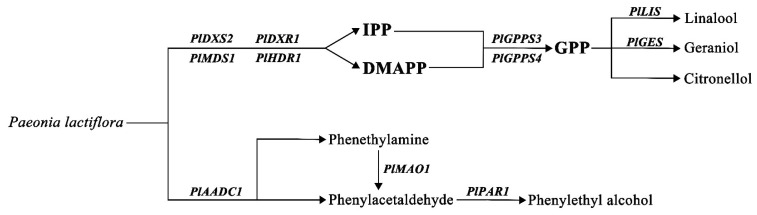
Synthetic pathway of characteristic aroma of herbaceous peony.

In summary, we conducted sensory evaluation on 87 herbaceous peony cultivars, and we found that there were only 16 cultivars with strong scent. The scarcity of strong-scented cultivars indicated the importance of herbaceous peony fragrance breeding. By analyzing the volatile compound emissions of 16 strong-scented cultivars, we found that the main volatile substances in herbaceous peony are terpenes, benzenoids/phenylpropanoids, and fatty acid derivatives, and we identified the characteristic aroma components of herbaceous peony as monoterpenes (citronellol, geraniol, and linalool) and 2-PE. Differences in the content and proportion of characteristic aroma components lead to different olfactory experiences. Based on the OAV of the aroma components, strong-scented cultivars were divided into three aroma types (a rose scent, a lily scent, and a mixed scent). In addition, we also studied the related genes in the synthetic pathway of characteristic aroma components and mapped the pathway map of herbaceous peony aroma release (Figure 9). These findings will provide key resources for the evaluation of herbaceous peony aroma volatiles and help in the selection of breeding materials based on the volatile components produced, leading to the development of more varieties of herbaceous peony with pleasant scent.

## Data Availability

Data are contained within the article or supplementary material.

## Supplementary Materials

The following supporting information can be downloaded at: https://www.mdpi.com/article/10.3390/ijms24119410/s1.
