# Supplementary material for: Volatile Composition and Classification of Paeonia lactiflora Flower Aroma Types and Identification of the Fragrance-Related Genes
Source: Int J Mol Sci. 2023 May 28;24(11):9410. doi: 10.3390/ijms24119410 (PMC10253308; doi:10.3390/ijms24119410)
Supplement: Supplementary file 1 [file ijms-24-09410-s001.zip › ijms-2386104-supplementary.pdf]

Table S1. Sensory evaluation scores of 87 herbaceous peony cultivars.

Cultivars	Group	Score (mean ± SD)	Pic.	Aroma level		Cultivars	Group	Score (mean ± SD)	Pic.	Aroma level
Dan Feng	Lactiflora peony cultivar group	3.913±0.042	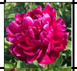	strong fragrance (3-4 points)		Jin Zan Ci Yu	Lactiflora peony cultivar group	1.958±0.088	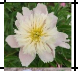	no/light fragrance (1-2 points)
Cang Long	Lactiflora peony cultivar group	3.788±0.061	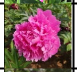	strong fragrance (3-4 points)		First Arrival	Itoh peony cultivar group	1.905±0.012	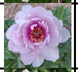	no/light fragrance (1-2 points)
Hong Cha Hua	Lactiflora peony cultivar group	3.712±0.087	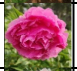	strong fragrance (3-4 points)		Kun Shan Xia Guang	Lactiflora peony cultivar group	1.890±0.054	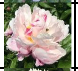	no/light fragrance (1-2 points)
Wu Hua Long Yu	Lactiflora peony cultivar group	3.614±0.077	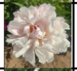	strong fragrance (3-4 points)		Sha Jin Guan Ding	Lactiflora peony cultivar group	1.814±0.073	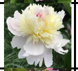	no/light fragrance (1-2 points)
Madame De Verneville	Lactiflora peony cultivar group	3.609±0.041	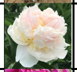	strong fragrance (3-4 points)		Pink Giant	Lactiflora peony cultivar group	1.811±0.105	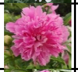	no/light fragrance (1-2 points)
Red Sarah Bernhardt	Lactiflora peony cultivar group	3.586±0.071	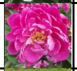	strong fragrance (3-4 points)		Huo Lian Jin Dan	Lactiflora peony cultivar group	1.801±0.076	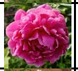	no/light fragrance (1-2 points)
Chi Fen	Lactiflora peony cultivar group	3.460±0.052	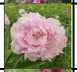	strong fragrance (3-4 points)		Feng Yu Luo Jin Chi	Lactiflora peony cultivar group	1.730±0.085	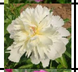	no/light fragrance (1-2 points)
Hei Xiu Qiu	Lactiflora peony cultivar group	3.299±0.061	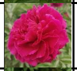	strong fragrance (3-4 points)		Kansas	Lactiflora peony cultivar group	1.729±0.066	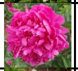	no/light fragrance (1-2 points)
Lollipop	Itoh peony cultivar group	3.271±0.066	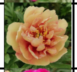	strong fragrance (3-4 points)		Jin Dai Wei	Lactiflora peony cultivar group	1.710±0.045	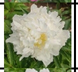	no/light fragrance (1-2 points)
Hong Feng Zhan Chi	Lactiflora peony cultivar group	3.250±0.105	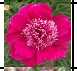	strong fragrance (3-4 points)		Xi Shi Lan	Lactiflora peony cultivar group	1.668±0.061	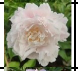	no/light fragrance (1-2 points)
Hong Xiu Qiu	Lactiflora peony cultivar group	3.163±0.034	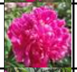	strong fragrance (3-4 points)		Hong Yan Fei Shuang	Lactiflora peony cultivar group	1.613±0.031	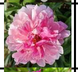	no/light fragrance (1-2 points)
Alexander Fleming	Lactiflora peony cultivar group	3.151±0.045	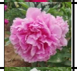	strong fragrance (3-4 points)		Yan Zi Xiang Yang	Lactiflora peony cultivar group	1.610±0.016	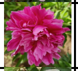	no/light fragrance (1-2 points)
Fen Yu Lou	Lactiflora peony cultivar group	3.102±0.013	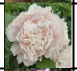	strong fragrance (3-4 points)		Callie's Memory	Itoh peony cultivar group	1.601±0.071	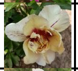	no/light fragrance (1-2 points)
Yuan Ye Jin Qiu	Lactiflora peony cultivar group	3.050±0.094	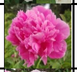	strong fragrance (3-4 points)		Cora Louise	Itoh peony cultivar group	1.598±0.023	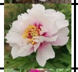	no/light fragrance (1-2 points)
Angel Cheeks	Lactiflora peony cultivar group	3.044±0.072	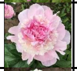	strong fragrance (3-4 points)		Paul M. Wild	Lactiflora peony cultivar group	1.579±0.096	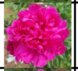	no/light fragrance (1-2 points)
Gardenia	Lactiflora peony cultivar group	3.002±0.035	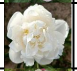	strong fragrance (3-4 points)		White Cap	Lactiflora peony cultivar group	1.546±0.077	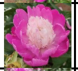	no/light fragrance (1-2 points)
Yang Fei Chu Yu	Lactiflora peony cultivar group	2.939±0.101	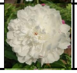	medium fragrance (2-3 points)		Qi Hua Lu Shuang	Lactiflora peony cultivar group	1.545±0.019	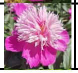	no/light fragrance (1-2 points)

Hong Guang Qi Jin	Lactiflora peony cultivar group	2.908±0.023	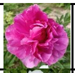	medium fragrance (2-3 points)		Hu Shui Dang Xia	Lactiflora peony cultivar group	1.528±0.071	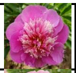	no/light fragrance (1-2 points)
Xiu Lan	Lactiflora peony cultivar group	2.882±0.019	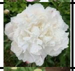	medium fragrance (2-3 points)		Shui Ying Chun Lan	Lactiflora peony cultivar group	1.522±0.082	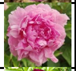	no/light fragrance (1-2 points)
Yellow Crown	Itoh peony cultivar group	2.871±0.083	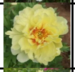	medium fragrance (2-3 points)		Zi Xia Ying Xue	Lactiflora peony cultivar group	1.501±0.052	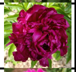	no/light fragrance (1-2 points)
Border charm	Itoh peony cultivar group	2.870±0.056	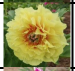	medium fragrance (2-3 points)		Karl Rosenfield	Lactiflora peony cultivar group	1.447±0.075	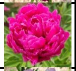	no/light fragrance (1-2 points)
Fu Shou Shuang Quan	Lactiflora peony cultivar group	2.834±0.044	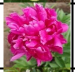	medium fragrance (2-3 points)		Mons. Jules Elie	Lactiflora peony cultivar group	1.417±0.069	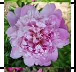	no/light fragrance (1-2 points)
Cheddar Cheese	Lactiflora peony cultivar group	2.827±0.031	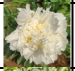	medium fragrance (2-3 points)		Hong Fu Shi	Lactiflora peony cultivar group	1.329±0.079	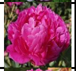	no/light fragrance (1-2 points)
Canary Brilliants	Itoh peony cultivar group	2.810±0.061	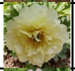	medium fragrance (2-3 points)		Hua Hong Chong Lou	Lactiflora peony cultivar group	1.319±0.067	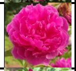	no/light fragrance (1-2 points)
Lian Tai	Lactiflora peony cultivar group	2.805±0.103	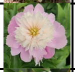	medium fragrance (2-3 points)		Red Magic	Lactiflora peony cultivar group	1.312±0.041	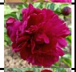	no/light fragrance (1-2 points)
Festiva Maxima	Lactiflora peony cultivar group	2.701±0.047	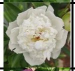	medium fragrance (2-3 points)		Buckeye Belle	Hybrid peony cultivar group	1.307±0.044	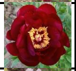	no/light fragrance (1-2 points)
Celebrity	Lactiflora peony cultivar group	2.657±0.088	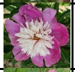	medium fragrance (2-3 points)		Susie Q	Lactiflora peony cultivar group	1.301±0.103	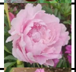	no/light fragrance (1-2 points)
Hillary	Itoh peony cultivar group	2.646±0.016	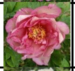	medium fragrance (2-3 points)		Jacorma	Lactiflora peony cultivar group	1.273±0.076	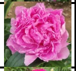	no/light fragrance (1-2 points)
Bing Qing	Lactiflora peony cultivar group	2.517±0.078	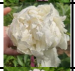	medium fragrance (2-3 points)		Ole Faithful	Hybrid peony cultivar group	1.234±0.105	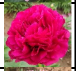	no/light fragrance (1-2 points)
Fen Fu Rong	Lactiflora peony cultivar group	2.477±0.091	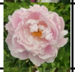	medium fragrance (2-3 points)		Rosea Plena	Lactiflora peony cultivar group	1.223±0.059	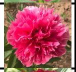	no/light fragrance (1-2 points)
Fen Chi Jin Yu	Lactiflora peony cultivar group	2.448±0.085	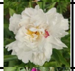	medium fragrance (2-3 points)		Da Di Jie Chun	Lactiflora peony cultivar group	1.222±0.074	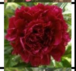	no/light fragrance (1-2 points)
Zi Feng Yu	Lactiflora peony cultivar group	2.407±0.029	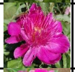	medium fragrance (2-3 points)		Pietertje Vriend Wagenaar	Lactiflora peony cultivar group	1.215±0.078	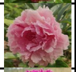	no/light fragrance (1-2 points)
Hong Yun Ying Ri	Lactiflora peony cultivar group	2.396±0.034	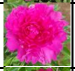	medium fragrance (2-3 points)		Tie Gan Zi	Lactiflora peony cultivar group	1.214±0.054	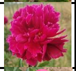	no/light fragrance (1-2 points)
Mo Zi Ling	Lactiflora peony cultivar group	2.388±0.079	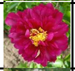	medium fragrance (2-3 points)		Coral Sunset	Hybrid peony cultivar group	1.202±0.023	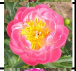	no/light fragrance (1-2 points)
Sarah Bernhardt	Lactiflora peony cultivar group	2.316±0.030	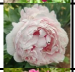	medium fragrance (2-3 points)		Adolphe Rousseau	Lactiflora peony cultivar group	1.179±0.049	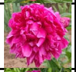	no/light fragrance (1-2 points)
Hong Feng	Lactiflora peony cultivar group	2.305±0.055	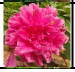	medium fragrance (2-3 points)		Da Hong Pao	Lactiflora peony cultivar group	1.176±0.048	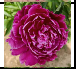	no/light fragrance (1-2 points)

Lan Ju	Lactiflora peony cultivar group	2.285±0.076	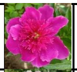	medium fragrance (2-3 points)		Mister Ed	Lactiflora peony cultivar group	1.177±0.064	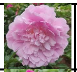	no/light fragrance (1-2 points)
Zhong Sheng Fen	Lactiflora peony cultivar group	2.275±0.066	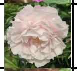	medium fragrance (2-3 points)		Ruth Clay	Lactiflora peony cultivar group	1.167±0.085	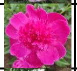	no/light fragrance (1-2 points)
Sai Fu Gui	Lactiflora peony cultivar group	2.208±0.075	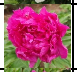	medium fragrance (2-3 points)		Fen Zhu Pan	Lactiflora peony cultivar group	1.138±0.058	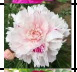	no/light fragrance (1-2 points)
Mei Gui Zi	Lactiflora peony cultivar group	2.194±0.046	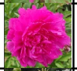	medium fragrance (2-3 points)		Lao Lai Hong	Lactiflora peony cultivar group	1.109±0.075	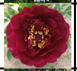	no/light fragrance (1-2 points)
Duchesse de Nemours	Lactiflora peony cultivar group	2.133±0.101	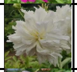	medium fragrance (2-3 points)		Tao Li Yan	Lactiflora peony cultivar group	1.098±0.039	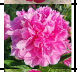	no/light fragrance (1-2 points)
Qing Wen	Lactiflora peony cultivar group	2.073±0.097	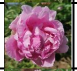	medium fragrance (2-3 points)		Red Charm	Hybrid peony cultivar group	1.094±0.090	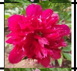	no/light fragrance (1-2 points)
Martha Bulloch	Lactiflora peony cultivar group	2.023±0.086	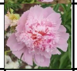	medium fragrance (2-3 points)		Hang Shao	Lactiflora peony cultivar group	1.052±0.072	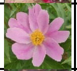	no/light fragrance (1-2 points)
						Joker	Lactiflora peony cultivar group	1.044±0.035	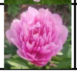	no/light fragrance (1-2 points)

Table S2. Sixty-eight compounds from 17 herbaceous peony cultivars.

Substances (Type)	RT (min)	Cultivar name and amount released (ng/g)																
		Alexander Fleming	Angel Cheeks	Cang Long	Chi Fen	Dan Feng	Fen Yu Lou	Gardenia	Hei Xiu Qiu	Hong Cha Hua	Hong Feng Zhan Chi	Hong Xiu Qiu	Lollipop	Madame De Verneville	Red Sarah Bernhardt	Wu Hua Long Yu	Yuan Ye Jin Qiu	Joker
**Terpenoids (41)**																		
**Monoterpenoids (28)**																		
α-Pinene	9.27	0.00±0.00	16.19±6.70	154.15±7.22	18.31±0.57	374.45±64.68	4.11±0.88	5.31±0.54	39.75±4.98	734.09±60.98	223.29±11.30	8.94±1.32	767.67±10.44	163.72±52.09	0.00±0.00	0.60±0.05	40.41±1.82	0.00±0.00
β-Pinene	11.15	0.00±0.00	0.00±0.00	259.26±12.34	28.56±1.64	0.00±0.00	0.00±0.00	0.00±0.00	0.00±0.00	0.00±0.00	0.00±0.00	0.00±0.00	0.00±0.00	0.00±0.00	150.29±19.35	0.00±0.00	0.00±0.00	0.00±0.00
α-Phellandrene	11.65	0.00±0.00	0.00±0.00	0.00±0.00	0.00±0.00	0.00±0.00	0.00±0.00	0.00±0.00	0.00±0.00	0.00±0.00	0.00±0.00	0.00±0.00	25.76±0.84	0.00±0.00	0.00±0.00	0.00±0.00	0.00±0.00	0.00±0.00
2-Carene	11.99	0.00±0.00	0.00±0.00	91.53±7.88	0.00±0.00	0.00±0.00	26.69±7.18	0.00±0.00	0.00±0.00	17.51±1.26	38.82±1.38	0.00±0.00	38.01±1.55	0.00±0.00	39.12±0.67	1.21±0.89	0.00±0.00	0.00±0.00
o-Cymene	12.25	4.10±1.23	0.00±0.00	0.00±0.00	0.00±0.00	79.42±1.31	9.77±1.53	0.00±0.00	1.67±0.45	24.43±1.46	7.05±1.08	0.00±0.00	16.09±3.54	0.00±0.00	36.86±1.26	0.00±0.00	0.00±0.00	0.00±0.00
D-Limonene	12.40	0.00±0.00	0.00±0.00	73.63±7.59	0.00±0.00	154.50±12.51	0.00±0.00	0.00±0.00	0.00±0.00	184.48±26.93	0.00±0.00	1.70±0.10	153.25±10.28	42.61±6.84	0.00±0.00	1.21±0.19	0.00±0.00	0.00±0.00
Eucalyptol	12.44	32.93±5.03	26.15±7.28	0.00±0.00	0.00±0.00	0.00±0.00	69.69±3.29	8.60±2.81	0.00±0.00	0.00±0.00	183.47±33.49	2.79±0.98	0.00±0.00	0.00±0.00	3.10±0.40	1.52±0.78	0.00±0.00	0.00±0.00
ç-Terpinene	13.30	0.00±0.00	0.00±0.00	0.00±0.00	0.00±0.00	0.00±0.00	6.82±0.29	0.00±0.00	0.00±0.00	13.62±2.71	0.00±0.00	0.00±0.00	18.11±4.47	0.00±0.00	33.54±0.95	0.00±0.00	0.00±0.00	0.00±0.00
Linalool oxide	13.69	0.00±0.00	7.06±1.50	0.00±0.00	0.00±0.00	0.00±0.00	0.00±0.00	3.16±0.68	0.00±0.00	0.00±0.00	0.00±0.00	0.00±0.00	23.79±3.16	22.99±6.04	21.13±3.97	0.00±0.00	0.00±0.00	0.00±0.00
Limonene	14.16	0.00±0.00	0.00±0.00	0.00±0.00	0.00±0.00	27.63±3.04	0.00±0.00	0.00±0.00	2.78±0.42	30.90±3.87	10.75±0.79	0.00±0.00	51.00±12.24	0.00±0.00	0.00±0.00	0.00±0.00	0.00±0.00	0.00±0.00
Linalool	14.62	0.00±0.00	803.29±24.04	164.00±5.24	119.35±6.15	185.75±9.51	0.00±0.00	609.27±23.64	169.29±18.50	173.45±17.58	375.22±25.13	751.69±8.56	1968.19±135.69	511.26±88.21	319.53±22.91	251.29±9.78	0.00±0.00	0.00±0.00
(-)-cis-rose oxide	14.91	0.00±0.00	0.00±0.00	0.00±0.00	13.88±3.23	43.14±6.44	0.00±0.00	0.00±0.00	1.55±0.39	0.00±0.00	8.62±1.16	0.00±0.00	0.00±0.00	12.48±0.52	17.47±4.55	0.00±0.00	0.00±0.00	0.00±0.00
(4e,6e)-alloocimene	15.46	0.00±0.00	0.00±0.00	0.00±0.00	0.00±0.00	0.00±0.00	0.00±0.00	0.00±0.00	0.00±0.00	27.85±3.44	0.00±0.00	0.00±0.00	45.19±9.67	0.00±0.00	34.43±0.98	0.00±0.00	0.00±0.00	0.00±0.00
(S)-(-)-Citronellal	16.17	0.00±0.00	0.00±0.00	0.00±0.00	13.33±2.47	85.56±5.60	0.00±0.00	0.00±0.00	0.00±0.00	1.48±0.47	0.00±0.00	0.00±0.00	0.00±0.00	0.00±0.00	0.00±0.00	0.00±0.00	0.00±0.00	0.00±0.00
trans-Verbenol	16.43	0.00±0.00	0.00±0.00	0.00±0.00	0.00±0.00	0.00±0.00	0.00±0.00	0.00±0.00	0.00±0.00	13.78±4.58	0.00±0.00	0.00±0.00	0.00±0.00	0.00±0.00	12.41±1.14	0.00±0.00	0.00±0.00	0.00±0.00
Lavandulol	16.56	0.00±0.00	0.00±0.00	0.00±0.00	28.25±2.70	0.00±0.00	0.00±0.00	0.00±0.00	0.00±0.00	0.00±0.00	0.00±0.00	0.00±0.00	0.00±0.00	0.00±0.00	0.00±0.00	0.00±0.00	0.00±0.00	0.00±0.00
Isoneral	16.99	0.00±0.00	0.00±0.00	0.00±0.00	0.00±0.00	0.00±0.00	0.00±0.00	0.00±0.00	0.00±0.00	33.74±0.43	0.00±0.00	0.00±0.00	0.00±0.00	21.55±2.87	14.05±2.76	0.00±0.00	0.00±0.00	0.00±0.00
Myrtenal	17.13	29.33±9.75	12.17±0.22	0.00±0.00	17.22±5.20	101.67±14.23	421.53±4.47	5.54±0.26	6.72±0.31	25.37±9.06	9.77±2.10	0.00±0.00	0.00±0.00	0.00±0.00	0.00±0.00	277.00±10.99	17.63±5.35	6.46±0.42
α-Terpineol	17.48	0.00±0.00	3.30±0.51	0.00±0.00	0.00±0.00	0.00±0.00	0.00±0.00	4.27±0.58	4.76±1.68	5.45±1.76	10.19±3.36	0.00±0.00	0.00±0.00	83.40±1.98	12.29±3.95	0.00±0.00	0.00±0.00	0.00±0.00
(1S)-Verbenone	17.85	0.00±0.00	0.00±0.00	339.70±50.82	0.00±0.00	0.00±0.00	61.76±7.61	0.00±0.00	0.00±0.00	0.00±0.00	0.00±0.00	0.00±0.00	0.00±0.00	0.00±0.00	0.00±0.00	0.00±0.00	0.00±0.00	0.00±0.00
(+)-β-citronellol	18.14	0.00±0.00	0.00±0.00	46.70±4.08	11.66±1.43	0.00±0.00	0.00±0.00	0.00±0.00	0.00±0.00	21.15±5.81	0.00±0.00	0.00±0.00	0.00±0.00	8.55±1.02	0.00±0.00	0.00±0.00	0.00±0.00	0.00±0.00
Nerol	18.39	0.00±0.00	0.00±0.00	0.00±0.00	0.00±0.00	0.00±0.00	113.14±2.84	0.00±0.00	0.00±0.00	0.00±0.00	0.00±0.00	0.00±0.00	0.00±0.00	0.00±0.00	0.00±0.00	0.00±0.00	0.00±0.00	4.81±0.27
Citronellol	18.52	111.37±15.69	0.00±0.00	3506.51±270.35	1315.70±42.26	8175.22±392.51	0.00±0.00	608.92±5.53	2502.31±179.43	480.58±32.26	1873.56±102.75	0.00±0.00	0.00±0.00	2250.67±124.41	1323.10±103.32	4696.01±200.19	0.00±0.00	0.00±0.00
p-Mentha-1(7),8(10)-dien-9-ol	18.89	0.00±0.00	0.00±0.00	0.00±0.00	0.00±0.00	0.00±0.00	29.93±7.09	0.00±0.00	0.00±0.00	0.00±0.00	0.00±0.00	0.00±0.00	0.00±0.00	0.00±0.00	0.00±0.00	0.00±0.00	0.00±0.00	0.00±0.00
Citral	18.72	113.20±15.70	0.00±0.00	294.58±21.47	184.31±25.00	480.68±27.65	0.00±0.00	0.00±0.00	167.95±5.83	876.35±119.18	244.25±16.26	467.44±14.41	128.86±5.08	973.82±85.26	577.52±45.70	266.47±40.67	131.04±11.13	0.00±0.00
Geraniol	19.13	108.46±1.65	0.00±0.00	641.02±67.74	145.09±15.59	554.58±22.03	114.49±2.01	411.09±14.36	102.98±10.29	1661.60±127.40	577.59±18.30	479.71±10.88	249.05±18.60	838.55±53.27	576.04±11.93	437.67±14.86	1193.35±63.20	0.00±0.00
(-)-cis-Myrtanol	19.43	0.00±0.00	0.00±0.00	0.00±0.00	0.00±0.00	0.00±0.00	262.89±21.77	0.00±0.00	0.00±0.00	0.00±0.00	0.00±0.00	0.00±0.00	0.00±0.00	0.00±0.00	0.00±0.00	2.43±0.82	0.00±0.00	0.00±0.00
perillyl aldehyde	19.74	0.00±0.00	0.00±0.00	0.00±0.00	0.00±0.00	0.00±0.00	45.18±7.76	0.00±0.00	0.00±0.00	0.00±0.00	0.00±0.00	0.00±0.00	0.00±0.00	0.00±0.00	0.00±0.00	15.54±1.87	11.51±0.89	0.00±0.00
**Sesquiterpenoids (10)**																		
Modephene	22.42	0.00±0.00	27.36±4.62	0.00±0.00	18.29±0.64	0.00±0.00	0.00±0.00	3.60±0.31	0.00±0.00	0.00±0.00	0.00±0.00	0.00±0.00	0.00±0.00	0.00±0.00	0.00±0.00	0.00±0.00	0.00±0.00	0.00±0.00
Alloaromadendrene	23.25	1.45±0.88	0.00±0.00	0.00±0.00	0.00±0.00	0.00±0.00	0.00±0.00	0.89±0.08	0.00±0.00	0.00±0.00	3.91±0.56	0.00±0.00	0.00±0.00	0.00±0.00	0.00±0.00	7.92±1.94	0.00±0.00	0.00±0.00
Caryophyllene	23.69	151.56±28.57	143.65±28.99	312.50±27.79	105.93±5.45	323.42±33.51	108.46±5.74	120.57±7.43	120.69±12.73	160.38±33.86	472.09±29.51	101.10±0.34	221.13±17.21	0.00±0.00	111.09±3.73	148.05±25.56	102.81±11.66	46.54±7.17
α-Longipinene	23.87	2.21±0.34	0.00±0.00	8.86±0.86	13.80±1.59	0.00±0.00	0.00±0.00	0.00±0.00	0.65±0.04	2.41±0.31	23.06±1.37	0.00±0.00	2.88±0.56	0.00±0.00	0.00±0.00	2.13±0.84	0.00±0.00	0.00±0.00
1R,3Z,9s-4,11,11-Trimethyl-8-methylenebicyclo[7.2.0]undec-3-ene	24.41	0.00±0.00	0.00±0.00	23.80±1.74	0.00±0.00	23.55±5.03	0.00±0.00	27.95±8.58	0.00±0.00	10.09±2.56	6.63±1.58	0.00±0.00	5.64±0.84	0.00±0.00	8.27±2.03	2.74±0.79	0.00±0.00	0.00±0.00
Humulene	24.57	17.62±1.99	8.40±0.62	36.97±2.42	0.00±0.00	68.97±2.70	16.41±5.45	2.81±0.26	2.78±0.19	10.84±1.17	58.39±18.34	0.00±0.00	60.18±2.90	0.00±0.00	0.00±0.00	18.58±3.36	10.62±1.36	9.23±0.77
Elemol	26.81	0.00±0.00	0.00±0.00	0.00±0.00	0.00±0.00	0.00±0.00	0.00±0.00	0.00±0.00	0.00±0.00	0.00±0.00	0.00±0.00	0.00±0.00	28.37±6.35	0.00±0.00	0.00±0.00	0.00±0.00	0.00±0.00	0.00±0.00
Nerolidol	27.09	0.00±0.00	8.46±0.41	0.00±0.00	0.00±0.00	0.00±0.00	0.00±0.00	23.34±1.99	0.00±0.00	8.22±0.36	5.86±0.13	8.59±1.61	41.50±2.67	45.01±4.59	45.54±5.96	0.00±0.00	0.00±0.00	0.00±0.00
Caryophyllene oxide	27.61	0.00±0.00	0.00±0.00	0.00±0.00	0.00±0.00	0.00±0.00	5.89±1.58	0.62±0.10	0.00±0.00	0.00±0.00	0.00±0.00	0.00±0.00	0.00±0.00	0.00±0.00	0.00±0.00	18.58±5.80	3.38±0.18	0.00±0.00
Caryophylla-4(12),8(13)-dien-5α-ol	29.36	3.50±0.24	0.00±0.00	0.00±0.00	0.00±0.00	20.86±1.64	0.00±0.00	2.50±0.39	0.75±0.02	2.22±0.11	0.00±0.00	0.00±0.00	93.50±13.11	0.00±0.00	0.00±0.00	0.00±0.00	0.00±0.00	0.00±0.00
**Irregular terpenoids (3)**																		
(1R)-(+)-Nopinone	15.75	0.00±0.00	0.00±0.00	0.00±0.00	0.00±0.00	0.00±0.00	87.51±16.96	0.00±0.00	0.00±0.00	0.00±0.00	0.00±0.00	0.00±0.00	0.00±0.00	0.00±0.00	0.00±0.00	47.84±10.81	0.00±0.00	0.00±0.00
Citronellyl acetate	21.76	0.00±0.00	0.00±0.00	0.00±0.00	0.00±0.00	70.06±3.97	0.00±0.00	0.00±0.00	16.59±4.98	0.00±0.00	0.00±0.00	0.00±0.00	0.00±0.00	137.80±11.73	0.00±0.00	89.59±28.33	0.00±0.00	0.00±0.00
Geranyl acetate	22.49	0.00±0.00	0.00±0.00	0.00±0.00	0.00±0.00	0.00±0.00	0.00±0.00	0.00±0.00	5.23±1.38	14.22±2.68	0.00±0.00	14.26±3.61	0.00±0.00	129.64±38.00	29.73±4.22	7.92±0.94	0.00±0.00	0.00±0.00
**Benzenoids/Phenylpropanoids (11)**																		
Benzaldehyde	10.25	0.00±0.00	0.00±0.00	0.00±0.00	61.44±3.86	0.00±0.00	18.76±6.96	0.00±0.00	1.73±0.27	0.00±0.00	0.00±0.00	0.00±0.00	0.00±0.00	23.73±5.81	5.30±0.92	0.00±0.00	0.00±0.00	23.56±3.91
Benzyl alcohol	12.62	0.00±0.00	0.00±0.00	0.00±0.00	89.06±11.93	0.00±0.00	52.33±9.15	0.00±0.00	0.00±0.00	0.00±0.00	0.00±0.00	0.00±0.00	0.00±0.00	7.23±0.89	0.00±0.00	1.52±0.19	0.00±0.00	47.17±1.78
Benzaldehyde, 2-hydroxy-	12.99	0.00±0.00	0.00±0.00	0.00±0.00	0.00±0.00	0.00±0.00	254.90±35.27	0.00±0.00	0.00±0.00	0.00±0.00	0.00±0.00	0.00±0.00	0.00±0.00	0.00±0.00	0.00±0.00	0.00±0.00	0.00±0.00	729.69±22.29
Acetophenone	13.53	0.00±0.00	0.00±0.00	0.00±0.00	27.59±5.81	0.00±0.00	0.00±0.00	0.00±0.00	0.00±0.00	39.71±17.75	0.00±0.00	0.00±0.00	0.00±0.00	0.00±0.00	26.83±3.10	0.00±0.00	0.00±0.00	0.00±0.00
Methyl benzoate	14.47	0.00±0.00	0.00±0.00	0.00±0.00	0.00±0.00	0.00±0.00	0.00±0.00	0.00±0.00	0.00±0.00	0.00±0.00	0.00±0.00	0.00±0.00	0.00±0.00	18.65±1.72	0.00±0.00	0.00±0.00	0.00±0.00	25.42±5.73
Phenylethyl Alcohol	15.15	1244.62±110.82	875.56±29.81	3932.51±236.98	611.83±59.90	116.59±10.11	1091.02±58.33	0.00±0.00	224.57±21.04	594.81±29.41	506.10±28.97	0.00±0.00	774.73±26.1	740.44±73.38	0.00±0.00	0.00±0.00	0.00±0.00	3.36±0.12
Benzeneacetic acid, 4-tridecyl ester	15.37	0.00±0.00	0.00±0.00	0.00±0.00	0.00±0.00	0.00±0.00	0.00±0.00	0.00±0.00	0.00±0.00	0.00±0.00	30.73±1.66	0.00±0.00	0.00±0.00	12.28±1.28	0.00±0.00	0.00±0.00	0.00±0.00	0.00±0.00
Benzene, 1,2-dimethoxy-	15.92	0.00±0.00	0.00±0.00	0.00±0.00	0.00±0.00	254.97±1.41	0.00±0.00	49.66±6.42	0.00±0.00	0.00±0.00	0.00±0.00	0.00±0.00	0.00±0.00	309.70±8.48	66.50±2.32	0.00±0.00	0.00±0.00	0.00±0.00
Benzene, 1,4-dimethoxy-	16.51	0.00±0.00	0.00±0.00	0.00±0.00	0.00±0.00	604.13±46.28	0.00±0.00	15.26±5.22	62.49±7.84	106.60±3.44	0.00±0.00	0.00±0.00	18.68±1.10	0.00±0.00	28.75±6.43	46.31±11.88	0.00±0.00	14.32±0.83
Methyl salicylate	17.46	0.00±0.00	0.00±0.00	0.00±0.00	0.00±0.00	0.00±0.00	0.00±0.00	0.00±0.00	0.00±0.00	0.00±0.00	0.00±0.00	0.00±0.00	0.00±0.00	0.00±0.00	0.00±0.00	0.00±0.00	0.00±0.00	540.23±42.78
Methyl cis-cinnamate	22.74	0.00±0.00	46.62±13.30	0.00±0.00	0.00±0.00	0.00±0.00	0.00±0.00	0.00±0.00	5.14±0.46	0.00±0.00	0.00±0.00	0.00±0.00	0.00±0.00	0.00±0.00	0.00±0.00	0.00±0.00	0.00±0.00	0.00±0.00
**Fatty acid derivatives (16)**																		
Hexanal	5.21	492.56±18.44	354.57±30.18	118.86±20.45	363.69±45.94	511.76±65.21	392.77±46.95	309.88±21.22	306.01±16.40	412.80±13.87	419.45±68.38	446.48±43.97	771.36±35.02	286.77±40.09	381.98±33.29	317.37±47.37	371.96±19.14	490.32±57.54
3-Hexenal	5.24	0.00±0.00	0.00±0.00	0.00±0.00	0.00±0.00	0.00±0.00	0.00±0.00	67.76±2.68	0.00±0.00	0.00±0.00	0.00±0.00	0.00±0.00	0.00±0.00	51.45±9.28	0.00±0.00	0.00±0.00	0.00±0.00	0.00±0.00
2-Hexyn-1-ol	6.50	15.17±0.82	0.00±0.00	0.00±0.00	10.71±1.77	18.66±1.88	6.67±1.98	0.00±0.00	0.81±0.11	3.05±0.91	0.00±0.00	0.00±0.00	0.00±0.00	0.00±0.00	13.41±2.53	0.00±0.00	0.00±0.00	0.00±0.00
2-Hexenal	6.85	723.97±83.11	568.46±80.67	273.50±35.89	251.45±20.76	523.62±59.85	365.33±18.68	596.78±17.95	223.11±43.52	436.45±16.38	723.61±168.07	389.61±57.06	517.48±116.35	815.93±139.11	383.63±73.06	616.20±37.04	849.79±61.15	674.49±29.70

3-Hexen-1-ol	6.89	128.08±12.63	122.74±8.53	544.70±89.44	233.10±8.40	117.07±19.85	192.41±16.94	156.73±12.24	282.63±13.77	222.16±11.60	0.00±0.00	233.54±27.08	0.00±0.00	194.86±19.33	239.59±18.93	177.66±18.67	0.00±0.00	0.00±0.00
trans-2-Hexenol	7.23	0.00±0.00	0.00±0.00	11.61±1.59	39.87±9.31	0.00±0.00	135.76±7.31	0.00±0.00	18.20±0.69	0.00±0.00	121.24±2.80	0.00±0.00	0.00±0.00	0.00±0.00	0.00±0.00	0.00±0.00	69.30±12.07	0.00±0.00
1-Hexanol	7.37	82.36±1.77	46.61±3.13	338.73±69.85	212.49±21.15	197.16±49.80	0.00±0.00	37.50±2.11	254.34±21.86	0.00±0.00	194.32±9.49	63.84±11.10	227.96±80.30	26.85±4.71	113.25±23.68	353.05±15.56	249.50±17.50	0.00±0.00
1-Hexanol, 2-ethyl-	12.40	0.00±0.00	0.00±0.00	0.00±0.00	100.29±7.37	0.00±0.00	0.00±0.00	0.00±0.00	0.00±0.00	0.00±0.00	0.00±0.00	0.00±0.00	0.00±0.00	0.00±0.00	165.39±28.16	0.00±0.00	0.00±0.00	0.00±0.00
Dodecane	13.77	0.00±0.00	0.00±0.00	0.00±0.00	0.00±0.00	0.00±0.00	0.00±0.00	0.00±0.00	0.00±0.00	0.00±0.00	27.30±4.05	0.00±0.00	0.00±0.00	0.00±0.00	3.60±0.76	0.00±0.00	87.80±6.57	0.00±0.00
2,6-Dimethyldecane	13.93	0.00±0.00	0.00±0.00	9.46±1.11	0.00±0.00	0.00±0.00	0.00±0.00	0.00±0.00	0.00±0.00	0.00±0.00	0.00±0.00	0.00±0.00	29.08±7.23	0.00±0.00	11.09±1.37	0.00±0.00	0.00±0.00	0.00±0.00
Undecane, 4-methyl-	14.39	0.00±0.00	0.00±0.00	26.60±4.26	0.00±0.00	0.00±0.00	0.00±0.00	0.00±0.00	0.00±0.00	0.00±0.00	14.63±1.56	0.00±0.00	0.00±0.00	0.00±0.00	0.00±0.00	0.00±0.00	0.00±0.00	0.00±0.00
Nonanal	14.74	2.04±0.11	4.53±0.51	0.00±0.00	25.56±0.36	24.88±6.39	0.00±0.00	0.00±0.00	5.90±0.56	0.00±0.00	0.00±0.00	1.71±0.33	0.00±0.00	11.59±0.29	33.05±3.70	0.00±0.00	0.00±0.00	7.71±0.97
trans-2-Nonenal	16.42	0.00±0.00	0.00±0.00	0.00±0.00	20.76±1.19	0.00±0.00	0.00±0.00	0.00±0.00	0.00±0.00	0.00±0.00	0.00±0.00	0.00±0.00	4.99±1.14	0.00±0.00	0.00±0.00	3.04±0.77	0.00±0.00	0.00±0.00
Heptadecane	30.30	0.00±0.00	2.77±0.29	0.00±0.00	0.00±0.00	0.00±0.00	0.00±0.00	0.00±0.00	0.00±0.00	0.00±0.00	0.00±0.00	12.47±2.26	0.00±0.00	12.45±0.70	0.00±0.00	0.00±0.00	31.21±8.57	27.91±5.89
2-Pentadecanone, 6,10,14-trimethyl-	33.29	8.00±0.49	4.23±0.69	21.13±2.67	3.98±0.61	0.00±0.00	0.00±0.00	3.82±0.91	0.00±0.00	0.00±0.00	0.00±0.00	5.22±0.41	0.00±0.00	13.18±0.73	2.53±0.31	0.00±0.00	1.74±0.33	4.93±0.98
Eicosane	39.04	6.49±1.59	4.74±0.76	21.07±7.09	7.41±2.04	47.37±6.73	14.12±5.49	4.30±0.67	13.76±1.64	28.43±1.79	15.29±5.04	8.01±0.90	6.39±0.22	16.57±4.70	6.39±0.65	0.00±0.00	18.39±6.83	17.49±1.24

Table S3. Thirty-two newly founded compounds from 17 cultivars.

Substances (Type)	RT (min)	Cultivar name and amount released (ng/g)																
		Alexander Fleming	Angel Cheeks	Cang Long	Chi Fen	Dan Feng	Fen Yu Lou	Gardenia	Hei Xiu Qiu	Hong Cha Hua	Hong Feng Zhan Chi	Hong Xiu Qiu	Lollipop	Madame De Verneville	Red Sarah Bernhardt	Wu Hua Long Yu	Yuan Ye Jin Qiu	Joker
**Terpenoids (13)**																		
**Monoterpenoids (8)**																		
2-Carene	11.99	0.00±0.00	0.00±0.00	0.00±0.00	0.00±0.00	0.00±0.00	26.69±7.18	0.00±0.00	0.00±0.00	17.51±1.26	7.82±1.38	0.00±0.00	38.01±1.55	0.00±0.00	8.55±0.67	1.21±0.89	0.00±0.00	0.00±0.00
o-Cymene	12.25	4.10±1.23	0.00±0.00	0.00±0.00	0.00±0.00	22.70±1.31	9.77±1.53	0.00±0.00	1.67±0.45	24.43±1.46	7.05±1.08	0.00±0.00	16.09±3.54	0.00±0.00	9.39±1.26	0.00±0.00	0.00±0.00	0.00±0.00
Linalool oxide	13.69	0.00±0.00	7.06±1.50	0.00±0.00	0.00±0.00	0.00±0.00	0.00±0.00	3.16±0.68	0.00±0.00	0.00±0.00	0.00±0.00	0.00±0.00	23.79±3.16	22.99±6.04	21.13±3.97	0.00±0.00	0.00±0.00	0.00±0.00
Lavandulol	16.56	0.00±0.00	0.00±0.00	0.00±0.00	28.25±2.70	0.00±0.00	0.00±0.00	0.00±0.00	0.00±0.00	0.00±0.00	0.00±0.00	0.00±0.00	0.00±0.00	0.00±0.00	0.00±0.00	0.00±0.00	0.00±0.00	0.00±0.00
Isoneral	16.99	0.00±0.00	0.00±0.00	0.00±0.00	0.00±0.00	0.00±0.00	0.00±0.00	0.00±0.00	0.00±0.00	33.74±0.43	0.00±0.00	0.00±0.00	0.00±0.00	21.55±2.87	14.05±2.76	0.00±0.00	0.00±0.00	0.00±0.00
Myrtenal	17.13	29.33±9.75	12.17±0.22	0.00±0.00	17.22±5.20	101.67±14.23	421.53±4.47	5.54±0.26	6.72±0.31	25.37±9.06	9.77±2.10	0.00±0.00	0.00±0.00	0.00±0.00	0.00±0.00	277.00±10.99	17.63±5.35	6.46±0.42
(-)-cis-Myrtanol	19.43	0.00±0.00	0.00±0.00	0.00±0.00	0.00±0.00	0.00±0.00	262.89±21.77	0.00±0.00	0.00±0.00	0.00±0.00	0.00±0.00	0.00±0.00	0.00±0.00	0.00±0.00	0.00±0.00	2.43±0.82	0.00±0.00	0.00±0.00
perillyl aldehyde	19.74	0.00±0.00	0.00±0.00	0.00±0.00	0.00±0.00	0.00±0.00	45.18±7.76	0.00±0.00	0.00±0.00	0.00±0.00	0.00±0.00	0.00±0.00	0.00±0.00	0.00±0.00	0.00±0.00	15.54±1.87	11.51±0.89	0.00±0.00
**Sesquiterpenoids (4)**																		
Modephene	22.42	0.00±0.00	27.36±4.62	0.00±0.00	18.29±0.64	0.00±0.00	0.00±0.00	3.60±0.31	0.00±0.00	0.00±0.00	0.00±0.00	0.00±0.00	0.00±0.00	0.00±0.00	0.00±0.00	0.00±0.00	0.00±0.00	0.00±0.00
Alloaromadendrene	23.25	1.45±0.88	0.00±0.00	0.00±0.00	0.00±0.00	0.00±0.00	0.00±0.00	0.89±0.08	0.00±0.00	0.00±0.00	3.91±0.56	0.00±0.00	0.00±0.00	0.00±0.00	0.00±0.00	7.92±1.94	0.00±0.00	0.00±0.00
Humulene	24.57	17.62±1.99	8.40±0.62	36.97±2.42	0.00±0.00	68.97±2.70	16.41±5.45	2.81±0.26	2.78±0.19	10.84±1.17	58.39±18.34	0.00±0.00	60.18±2.90	0.00±0.00	0.00±0.00	18.58±3.36	10.62±1.36	9.23±0.77
Elemol	26.81	0.00±0.00	0.00±0.00	0.00±0.00	0.00±0.00	0.00±0.00	0.00±0.00	0.00±0.00	0.00±0.00	0.00±0.00	0.00±0.00	0.00±0.00	28.37±6.35	0.00±0.00	0.00±0.00	0.00±0.00	0.00±0.00	0.00±0.00
**Irregular terpenoids (1)**																		
(1R)-(+)-Nopinone	15.75	0.00±0.00	0.00±0.00	0.00±0.00	0.00±0.00	0.00±0.00	87.51±16.96	0.00±0.00	0.00±0.00	0.00±0.00	0.00±0.00	0.00±0.00	0.00±0.00	0.00±0.00	0.00±0.00	47.84±10.81	0.00±0.00	0.00±0.00
**Benzenoids/Phenylpropanoids (7)**																		
Benzaldehyde	10.25	0.00±0.00	0.00±0.00	0.00±0.00	61.44±3.86	0.00±0.00	18.76±6.96	0.00±0.00	1.73±0.27	0.00±0.00	0.00±0.00	0.00±0.00	0.00±0.00	23.73±5.81	5.30±0.92	0.00±0.00	0.00±0.00	23.56±3.91
Benzyl alcohol	12.62	0.00±0.00	0.00±0.00	0.00±0.00	89.06±11.93	0.00±0.00	52.33±9.15	0.00±0.00	0.00±0.00	0.00±0.00	0.00±0.00	0.00±0.00	0.00±0.00	7.23±0.89	0.00±0.00	1.52±0.19	0.00±0.00	47.17±1.78
Benzaldehyde, 2-hydroxy-	12.99	0.00±0.00	0.00±0.00	0.00±0.00	0.00±0.00	0.00±0.00	254.90±35.27	0.00±0.00	0.00±0.00	0.00±0.00	0.00±0.00	0.00±0.00	0.00±0.00	0.00±0.00	0.00±0.00	0.00±0.00	0.00±0.00	729.69±22.29
Acetophenone	13.53	0.00±0.00	0.00±0.00	0.00±0.00	27.59±5.81	0.00±0.00	0.00±0.00	0.00±0.00	0.00±0.00	39.71±17.75	0.00±0.00	0.00±0.00	0.00±0.00	0.00±0.00	26.83±3.10	0.00±0.00	0.00±0.00	0.00±0.00
Methyl benzoate	14.47	0.00±0.00	0.00±0.00	0.00±0.00	0.00±0.00	0.00±0.00	0.00±0.00	0.00±0.00	0.00±0.00	0.00±0.00	0.00±0.00	0.00±0.00	0.00±0.00	18.65±1.72	0.00±0.00	0.00±0.00	0.00±0.00	25.42±5.73
Benzeneacetic acid, 4-tridecyl ester	15.37	0.00±0.00	0.00±0.00	0.00±0.00	0.00±0.00	0.00±0.00	0.00±0.00	0.00±0.00	0.00±0.00	0.00±0.00	30.73±1.66	0.00±0.00	0.00±0.00	12.28±1.28	0.00±0.00	0.00±0.00	0.00±0.00	0.00±0.00
Benzene, 1,2-dimethoxy-	15.92	0.00±0.00	0.00±0.00	0.00±0.00	0.00±0.00	254.97±1.41	0.00±0.00	49.66±6.42	0.00±0.00	0.00±0.00	0.00±0.00	0.00±0.00	0.00±0.00	309.70±8.48	66.50±2.32	0.00±0.00	0.00±0.00	0.00±0.00
**Fatty acid derivatives (12)**																		
Hexanal	5.21	492.56±18.44	354.57±30.18	118.86±20.45	363.69±45.94	511.76±65.21	392.77±46.95	309.88±21.22	306.01±16.40	412.80±13.87	419.45±68.38	446.48±43.97	771.36±35.02	286.77±40.09	381.98±33.29	317.37±47.37	371.96±19.14	490.32±57.54
3-Hexenal	5.24	0.00±0.00	0.00±0.00	0.00±0.00	0.00±0.00	0.00±0.00	0.00±0.00	67.76±2.68	0.00±0.00	0.00±0.00	0.00±0.00	0.00±0.00	0.00±0.00	51.45±9.28	0.00±0.00	0.00±0.00	0.00±0.00	0.00±0.00
2-Hexenal	6.85	723.97±83.11	568.46±80.67	273.50±35.89	251.45±20.76	523.62±59.85	365.33±18.68	596.78±17.95	223.11±43.52	436.45±16.38	723.61±168.07	389.61±57.06	517.48±116.35	815.93±139.11	383.63±73.06	616.20±37.04	849.79±61.15	674.49±29.70
trans-2-Hexenol	7.23	0.00±0.00	0.00±0.00	11.61±1.59	39.87±9.31	0.00±0.00	135.76±7.31	0.00±0.00	18.20±0.69	0.00±0.00	121.24±2.80	0.00±0.00	0.00±0.00	0.00±0.00	0.00±0.00	0.00±0.00	69.30±12.07	0.00±0.00
1-Hexanol, 2-ethyl-	12.40	0.00±0.00	0.00±0.00	0.00±0.00	100.29±7.37	0.00±0.00	0.00±0.00	0.00±0.00	0.00±0.00	0.00±0.00	0.00±0.00	0.00±0.00	0.00±0.00	0.00±0.00	165.39±28.16	0.00±0.00	0.00±0.00	0.00±0.00
Dodecane	13.77	0.00±0.00	0.00±0.00	0.00±0.00	0.00±0.00	0.00±0.00	0.00±0.00	0.00±0.00	0.00±0.00	0.00±0.00	27.30±4.05	0.00±0.00	0.00±0.00	0.00±0.00	3.60±0.76	0.00±0.00	87.80±6.57	0.00±0.00
2,6-Dimethyldecane	13.93	0.00±0.00	0.00±0.00	9.46±1.11	0.00±0.00	0.00±0.00	0.00±0.00	0.00±0.00	0.00±0.00	0.00±0.00	0.00±0.00	0.00±0.00	29.08±7.23	0.00±0.00	11.09±1.37	0.00±0.00	0.00±0.00	0.00±0.00
Undecane, 4-methyl-	14.39	0.00±0.00	0.00±0.00	26.60±4.26	0.00±0.00	0.00±0.00	0.00±0.00	0.00±0.00	0.00±0.00	0.00±0.00	14.63±1.56	0.00±0.00	0.00±0.00	0.00±0.00	0.00±0.00	0.00±0.00	0.00±0.00	0.00±0.00
Nonanal	14.74	2.04±0.11	4.53±0.51	0.00±0.00	25.56±0.36	24.88±6.39	0.00±0.00	0.00±0.00	5.90±0.56	0.00±0.00	0.00±0.00	1.71±0.33	0.00±0.00	11.59±0.29	33.05±3.70	0.00±0.00	0.00±0.00	7.71±0.97
trans-2-Nonenal	16.42	0.00±0.00	0.00±0.00	0.00±0.00	20.76±1.19	0.00±0.00	0.00±0.00	0.00±0.00	0.00±0.00	0.00±0.00	0.00±0.00	0.00±0.00	4.99±1.14	0.00±0.00	0.00±0.00	3.04±0.77	0.00±0.00	0.00±0.00
Heptadecane	30.30	0.00±0.00	2.77±0.29	0.00±0.00	0.00±0.00	0.00±0.00	0.00±0.00	0.00±0.00	0.00±0.00	0.00±0.00	0.00±0.00	12.47±2.26	0.00±0.00	12.45±0.70	0.00±0.00	0.00±0.00	31.21±8.57	27.91±5.89
Eicosane	39.04	6.49±1.59	4.74±0.76	21.07±7.09	7.41±2.04	47.37±6.73	14.12±5.49	4.30±0.67	13.76±1.64	28.43±1.79	15.29±5.04	8.01±0.90	6.39±0.22	16.57±4.70	6.39±0.65	0.00±0.00	18.39±6.83	17.49±1.24

Table S4. Compounds with a relative content greater than 100 ng/g among 17 cultivars.

Substances (Type)	RT (min)	Cultivar name and amount released (ng/g)																
		Alexander Fleming	Angel Cheeks	Cang Long	Chi Fen	Dan Feng	Fen Yu Lou	Gardenia	Hei Xiu Qiu	Hong Cha Hua	Hong Feng Zhan Chi	Hong Xiu Qiu	Lollipop	Madame De Verneville	Red Sarah Bernhardt	Wu Hua Long Yu	Yuan Ye Jin Qiu	Joker
**Terpenoids (15)**																		
**Monoterpenoids (12)**																		
α-Pinene	9.27	0.00±0.00	0.00±0.00	154.15±7.22	0.00±0.00	374.45±64.68	0.00±0.00	0.00±0.00	0.00±0.00	734.09±60.98	223.29±11.30	0.00±0.00	767.67±10.44	163.72±52.09	0.00±0.00	0.00±0.00	0.00±0.00	0.00±0.00
β-Pinene	11.15	0.00±0.00	0.00±0.00	259.26±12.34	0.00±0.00	0.00±0.00	0.00±0.00	0.00±0.00	0.00±0.00	0.00±0.00	0.00±0.00	0.00±0.00	0.00±0.00	0.00±0.00	150.29±19.35	0.00±0.00	0.00±0.00	0.00±0.00
D-Limonene	12.40	0.00±0.00	0.00±0.00	0.00±0.00	0.00±0.00	154.50±12.51	0.00±0.00	0.00±0.00	0.00±0.00	184.48±26.93	0.00±0.00	0.00±0.00	153.25±10.28	0.00±0.00	0.00±0.00	0.00±0.00	0.00±0.00	0.00±0.00
Eucalyptol	12.44	0.00±0.00	0.00±0.00	0.00±0.00	0.00±0.00	0.00±0.00	0.00±0.00	0.00±0.00	0.00±0.00	0.00±0.00	183.47±33.49	0.00±0.00	0.00±0.00	0.00±0.00	0.00±0.00	0.00±0.00	0.00±0.00	0.00±0.00
Linalool	14.62	0.00±0.00	803.29±24.04	164.00±5.24	119.35±6.15	185.75±9.51	0.00±0.00	609.27±23.64	169.29±18.50	173.45±17.58	375.22±25.13	751.69±8.56	1968.19±135.69	511.26±88.21	319.53±22.91	251.29±9.78	0.00±0.00	0.00±0.00
Myrtenal	17.13	0.00±0.00	0.00±0.00	0.00±0.00	0.00±0.00	101.67±14.23	421.53±4.47	0.00±0.00	0.00±0.00	0.00±0.00	0.00±0.00	0.00±0.00	0.00±0.00	0.00±0.00	0.00±0.00	277.00±10.99	0.00±0.00	0.00±0.00
(1S)-Verbenone	17.85	0.00±0.00	0.00±0.00	339.70±50.82	0.00±0.00	0.00±0.00	0.00±0.00	0.00±0.00	0.00±0.00	0.00±0.00	0.00±0.00	0.00±0.00	0.00±0.00	0.00±0.00	0.00±0.00	0.00±0.00	0.00±0.00	0.00±0.00
Nerol	18.39	0.00±0.00	0.00±0.00	0.00±0.00	0.00±0.00	0.00±0.00	113.14±2.84	0.00±0.00	0.00±0.00	0.00±0.00	0.00±0.00	0.00±0.00	0.00±0.00	0.00±0.00	0.00±0.00	0.00±0.00	0.00±0.00	0.00±0.00
Citronellol	18.52	111.37±15.69	0.00±0.00	3506.51±270.35	1315.70±42.26	8175.22±392.51	0.00±0.00	608.92±5.53	2502.31±179.43	480.58±32.26	1873.56±102.75	0.00±0.00	0.00±0.00	2250.67±124.41	1323.10±103.32	4696.01±200.19	0.00±0.00	0.00±0.00
Citral	18.72	113.20±15.70	0.00±0.00	294.58±21.47	184.31±25.00	480.68±27.65	0.00±0.00	0.00±0.00	167.95±5.83	876.35±119.18	244.25±16.26	467.44±14.41	128.86±5.08	973.82±85.26	577.52±45.70	266.47±40.67	131.04±11.13	0.00±0.00
Geraniol	19.13	108.46±1.65	0.00±0.00	641.02±67.74	145.09±15.59	554.58±22.03	114.49±2.01	411.09±14.36	102.98±10.29	1661.60±127.40	577.59±18.30	479.71±10.88	249.05±18.60	838.55±53.27	576.04±11.93	437.67±14.86	1193.35±63.20	0.00±0.00
(-)-cis-Myrtanol	19.43	0.00±0.00	0.00±0.00	0.00±0.00	0.00±0.00	0.00±0.00	262.89±21.77	0.00±0.00	0.00±0.00	0.00±0.00	0.00±0.00	0.00±0.00	0.00±0.00	0.00±0.00	0.00±0.00	0.00±0.00	0.00±0.00	0.00±0.00
**Sesquiterpenoids (1)**																		
Caryophyllene	23.69	151.56±28.57	143.65±28.99	312.50±27.79	105.93±5.45	323.42±33.51	108.46±5.74	120.57±7.43	120.69±12.73	160.38±33.86	472.09±29.51	101.10±0.34	221.13±17.21	0.00±0.00	111.09±3.73	148.05±25.56	102.81±11.66	0.00±0.00
**Irregular terpenoids (2)**																		
Citronellyl acetate	21.76	0.00±0.00	0.00±0.00	0.00±0.00	0.00±0.00	0.00±0.00	0.00±0.00	0.00±0.00	0.00±0.00	0.00±0.00	0.00±0.00	0.00±0.00	0.00±0.00	137.80±11.73	0.00±0.00	0.00±0.00	0.00±0.00	0.00±0.00
Geranyl acetate	22.49	0.00±0.00	0.00±0.00	0.00±0.00	0.00±0.00	0.00±0.00	0.00±0.00	0.00±0.00	0.00±0.00	0.00±0.00	0.00±0.00	0.00±0.00	0.00±0.00	129.64±38.00	0.00±0.00	0.00±0.00	0.00±0.00	0.00±0.00
**Benzenoids/Phenylpropanoids (5)**																		
Benzaldehyde, 2-hydroxy-	12.99	0.00±0.00	0.00±0.00	0.00±0.00	0.00±0.00	0.00±0.00	254.90±35.27	0.00±0.00	0.00±0.00	0.00±0.00	0.00±0.00	0.00±0.00	0.00±0.00	0.00±0.00	0.00±0.00	0.00±0.00	0.00±0.00	729.69±22.29
Phenylethyl Alcohol	15.15	1244.62±110.82	875.56±29.81	3932.51±236.98	611.83±59.90	116.59±10.11	1091.02±58.33	0.00±0.00	224.57±21.04	594.81±29.41	506.10±28.97	0.00±0.00	774.73±26.1	740.44±73.38	0.00±0.00	0.00±0.00	0.00±0.00	0.00±0.00
Benzene, 1,2-dimethoxy-	15.92	0.00±0.00	0.00±0.00	0.00±0.00	0.00±0.00	254.97±1.41	0.00±0.00	0.00±0.00	0.00±0.00	0.00±0.00	0.00±0.00	0.00±0.00	0.00±0.00	309.70±8.48	0.00±0.00	0.00±0.00	0.00±0.00	0.00±0.00
Benzene, 1,4-dimethoxy-	16.51	0.00±0.00	0.00±0.00	0.00±0.00	0.00±0.00	604.13±46.28	0.00±0.00	0.00±0.00	0.00±0.00	106.60±3.44	0.00±0.00	0.00±0.00	0.00±0.00	0.00±0.00	0.00±0.00	0.00±0.00	0.00±0.00	0.00±0.00
Methyl salicylate	17.46	0.00±0.00	0.00±0.00	0.00±0.00	0.00±0.00	0.00±0.00	0.00±0.00	0.00±0.00	0.00±0.00	0.00±0.00	0.00±0.00	0.00±0.00	0.00±0.00	0.00±0.00	0.00±0.00	0.00±0.00	0.00±0.00	540.23±42.78
**Fatty acid derivatives (6)**																		
Hexanal	5.21	492.56±18.44	354.57±30.18	118.86±20.45	363.69±45.94	511.76±65.21	392.77±46.95	309.88±21.22	306.01±16.40	412.80±13.87	419.45±68.38	446.48±43.97	771.36±35.02	286.77±40.09	381.98±33.29	317.37±47.37	371.96±19.14	490.32±57.54
2-Hexenal	6.85	723.97±83.11	568.46±80.67	273.50±35.89	251.45±20.76	523.62±59.85	365.33±18.68	596.78±17.95	223.11±43.52	436.45±16.38	723.61±168.07	389.61±57.06	517.48±116.35	815.93±139.11	383.63±73.06	616.20±37.04	849.79±61.15	674.49±29.70
3-Hexen-1-ol	6.89	128.08±12.63	122.74±8.53	544.70±89.44	233.10±8.40	117.07±19.85	192.41±16.94	156.73±12.24	282.63±13.77	222.16±11.60	0.00±0.00	233.54±27.08	0.00±0.00	194.86±19.33	239.59±18.93	177.66±18.67	0.00±0.00	0.00±0.00
trans-2-Hexenol	7.23	0.00±0.00	0.00±0.00	0.00±0.00	0.00±0.00	0.00±0.00	135.76±7.31	0.00±0.00	0.00±0.00	0.00±0.00	121.24±2.80	0.00±0.00	0.00±0.00	0.00±0.00	0.00±0.00	0.00±0.00	0.00±0.00	0.00±0.00
1-Hexanol	7.37	0.00±0.00	0.00±0.00	338.73±69.85	212.49±21.15	197.16±49.80	0.00±0.00	0.00±0.00	254.34±21.86	0.00±0.00	194.32±9.49	0.00±0.00	227.96±80.30	0.00±0.00	113.25±23.68	353.05±15.56	249.50±17.50	0.00±0.00
1-Hexanol, 2-ethyl-	12.40	0.00±0.00	0.00±0.00	0.00±0.00	100.29±7.37	0.00±0.00	0.00±0.00	0.00±0.00	0.00±0.00	0.00±0.00	0.00±0.00	0.00±0.00	0.00±0.00	0.00±0.00	165.39±28.16	0.00±0.00	0.00±0.00	0.00±0.00

Table S5. Descriptions and threshold values of major herbaceous peony scent compounds.

Substances (Type)	Odor discription	Odor Classification	Odor Threshold (mg/kg)	Odor Activity Value																
				Alexander Fleming	Angel Cheeks	Cang Long	Chi Fen	Dan Feng	Fen Yu Lou	Gardenia	Hei Xiu Qiu	Hong Cha Hua	Hong Feng Zhan Chi	Hong Xiu Qiu	Lollipop	Madame De Verneville	Red Sarah Bernhardt	Wu Hua Long Yu	Yuan Ye Jin Qiu	Joker
**Terpenoids (15)**																				
**Monoterpenoids (12)**																				
α-Pinene	Charastristic odor of pine	Herbal	0.12	0.00±0.00	0.00±0.00	1.28±0.06	0.00±0.00	3.12±0.54	0.00±0.00	0.00±0.00	0.00±0.00	6.12±0.51	1.86±0.09	0.00±0.00	6.40±0.09	1.36±0.43	0.00±0.00	0.00±0.00	0.00±0.00	0.00±0.00
β-Pinene	Characteristic turpentine odor with a dry, woody or resinous aroma	Woody	0.14	0.00±0.00	0.00±0.00	1.85±0.09	0.00±0.00	0.00±0.00	0.00±0.00	0.00±0.00	0.00±0.00	0.00±0.00	0.00±0.00	0.00±0.00	0.00±0.00	0.00±0.00	1.07±0.14	0.00±0.00	0.00±0.00	0.00±0.00
D-Limonene	Pleasant, lemon-like odor	Fruity-Citrus	0.034	0.00±0.00	0.00±0.00	0.00±0.00	0.00±0.00	4.54±0.37	0.00±0.00	0.00±0.00	0.00±0.00	5.43±0.79	0.00±0.00	0.00±0.00	4.51±0.30	0.00±0.00	0.00±0.00	0.00±0.00	0.00±0.00	0.00±0.00
Eucalyptol	Characteristic camphoraceous odor and fresh, pungent, cooling taste	Spicy	0.01	0.00±0.00	0.00±0.00	0.00±0.00	0.00±0.00	0.00±0.00	0.00±0.00	0.00±0.00	0.00±0.00	0.00±0.00	18.35±3.35	0.00±0.00	0.00±0.00	0.00±0.00	0.00±0.00	0.00±0.00	0.00±0.00	0.00±0.00
Linalool	Typical pleasant floral and citrus odor	Floral-lily	0.0015	0.00±0.00	535.53±16.03	109.33±3.49	79.57±4.10	123.83±6.34	0.00±0.00	406.18±15.76	112.86±12.33	115.63±11.72	250.15±16.75	501.13±5.71	1312.13±90.46	340.84±58.81	213.02±15.27	167.53±6.52	0.00±0.00	0.00±0.00
Myrtenal	Cinnamon odor	Spicy	n/a	0.00±0.00	0.00±0.00	0.00±0.00	0.00±0.00	n/a	n/a	0.00±0.00	0.00±0.00	0.00±0.00	0.00±0.00	0.00±0.00	0.00±0.00	0.00±0.00	0.00±0.00	n/a	0.00±0.00	0.00±0.00
(1S)-Verbenone	Verbena odor	Herbal	n/a	0.00±0.00	0.00±0.00	n/a	0.00±0.00	0.00±0.00	0.00±0.00	0.00±0.00	0.00±0.00	0.00±0.00	0.00±0.00	0.00±0.00	0.00±0.00	0.00±0.00	0.00±0.00	0.00±0.00	0.00±0.00	0.00±0.00
Nerol	Lemon-like, floral odor	Fruity-Citrus	0.3	0.00±0.00	0.00±0.00	0.00±0.00	0.00±0.00	0.00±0.00	0.38±0.01	0.00±0.00	0.00±0.00	0.00±0.00	0.00±0.00	0.00±0.00	0.00±0.00	0.00±0.00	0.00±0.00	0.00±0.00	0.00±0.00	0.00±0.00
Citronellol	Fresh rosy odor	Floral-rose	0.01	11.14±1.57	0.00±0.00	350.65±27.04	131.57±4.23	817.52±39.25	0.00±0.00	60.89±0.55	250.23±17.94	48.06±3.23	187.36±10.28	0.00±0.00	0.00±0.00	225.07±12.44	132.31±10.33	469.60±20.02	0.00±0.00	0.00±0.00
Citral	Citrus and lemon-like odor	Fruity-Citrus	0.032	3.54±0.49	0.00±0.00	9.21±0.67	5.76±0.78	15.02±0.86	0.00±0.00	0.00±0.00	5.25±0.18	27.39±3.72	7.63±0.51	14.61±0.45	4.03±0.16	30.43±2.66	18.05±1.43	8.33±1.27	4.10±0.35	0.00±0.00
Geraniol	Rose-like, sweet odor	Floral-rose	0.0075	14.46±0.22	0.00±0.00	85.47±9.03	19.35±2.08	73.94±2.94	15.27±0.27	54.81±1.91	13.73±1.37	221.55±16.99	77.01±2.44	63.96±1.45	33.21±2.48	111.81±7.10	76.81±1.59	58.36±1.98	159.11±8.43	0.00±0.00
(-)-cis-Myrtanol	Sandalwood and cool mint, soft odor	Woody	n/a	0.00±0.00	0.00±0.00	0.00±0.00	0.00±0.00	0.00±0.00	n/a	0.00±0.00	0.00±0.00	0.00±0.00	0.00±0.00	0.00±0.00	0.00±0.00	0.00±0.00	0.00±0.00	0.00±0.00	0.00±0.00	0.00±0.00
**Sesquiterpenoids (1)**																				
Caryophyllene	Woody-spicy, dry, clove-like odor	Woody	0.15	1.01±0.19	0.96±0.19	2.08±0.19	0.71±0.04	2.16±0.22	0.72±0.04	0.80±0.05	0.80±0.08	1.07±0.23	3.15±0.20	0.67±0.002	1.47±0.11	0.00±0.00	0.74±0.02	0.99±0.17	0.69±0.08	0.00±0.00
**Irregular terpenoids (2)**																				
Citronellyl acetate	Fresh, rose, fruity odor	Floral-rose	1	0.00±0.00	0.00±0.00	0.00±0.00	0.00±0.00	0.00±0.00	0.00±0.00	0.00±0.00	0.00±0.00	0.00±0.00	0.00±0.00	0.00±0.00	0.00±0.00	0.14±0.01	0.00±0.00	0.00±0.00	0.00±0.00	0.00±0.00
Geranyl acetate	Lavender odor	Herbal	0.1	0.00±0.00	0.00±0.00	0.00±0.00	0.00±0.00	0.00±0.00	0.00±0.00	0.00±0.00	0.00±0.00	0.00±0.00	0.00±0.00	0.00±0.00	0.00±0.00	1.30±0.38	0.00±0.00	0.00±0.00	0.00±0.00	0.00±0.00
**Benzenoids/Phenylpropanoids (5)**																				
Benzaldehyde, 2-hydroxy-	Almond and herb-like odor	Herbal	0.34	0.00±0.00	0.00±0.00	0.00±0.00	0.00±0.00	0.00±0.00	0.75±0.10	0.00±0.00	0.00±0.00	0.00±0.00	0.00±0.00	0.00±0.00	0.00±0.00	0.00±0.00	0.00±0.00	0.00±0.00	0.00±0.00	2.15±0.07
Phenylethyl Alcohol	Honey-like, floral odor	Floral-rose	0.045	27.66±2.46	19.46±0.66	87.39±5.27	13.60±1.33	2.59±0.22	24.24±1.30	0.00±0.00	4.99±0.47	13.22±0.65	11.25±0.64	0.00±0.00	17.22±0.58	16.45±1.63	0.00±0.00	0.00±0.00	0.00±0.00	0.00±0.00
Benzene, 1,2-dimethoxy-	Fruity odor	Fruity-sweet	0.775	0.00±0.00	0.00±0.00	0.00±0.00	0.00±0.00	0.33±0.002	0.00±0.00	0.00±0.00	0.00±0.00	0.00±0.00	0.00±0.00	0.00±0.00	0.00±0.00	0.40±0.01	0.00±0.00	0.00±0.00	0.00±0.00	0.00±0.00
Benzene, 1,4-dimethoxy-	Clove-like odor	Woody	0.0214	0.00±0.00	0.00±0.00	0.00±0.00	0.00±0.00	28.23±2.16	0.00±0.00	0.00±0.00	0.00±0.00	4.98±0.16	0.00±0.00	0.00±0.00	0.00±0.00	0.00±0.00	0.00±0.00	0.00±0.00	0.00±0.00	0.00±0.00
Methyl salicylate	Minty, spicy, sweet, wintergreen-like odor	Spicy	0.04	0.00±0.00	0.00±0.00	0.00±0.00	0.00±0.00	0.00±0.00	0.00±0.00	0.00±0.00	0.00±0.00	0.00±0.00	0.00±0.00	0.00±0.00	0.00±0.00	0.00±0.00	0.00±0.00	0.00±0.00	0.00±0.00	13.51±1.07
**Fatty acid derivatives (6)**																				
Hexanal	Grassy-green odor, apple odor	Herbal	0.0615	8.01±0.30	5.77±0.49	1.93±0.33	5.91±0.75	8.32±1.06	6.39±0.76	5.04±0.35	4.98±0.27	6.71±0.23	6.82±1.11	7.26±0.71	12.54±0.57	4.66±0.65	6.21±0.54	5.16±0.77	6.05±0.31	7.97±0.94
2-Hexenal	Leaves, fruits, vegetables-like odor	Herbal	0.3	2.41±0.28	1.89±0.27	0.91±0.12	0.84±0.07	1.75±0.20	1.22±0.06	1.99±0.06	0.74±0.15	1.45±0.05	2.41±0.56	1.30±0.19	1.72±0.39	2.72±0.46	1.28±0.24	2.05±0.12	2.83±0.20	2.25±0.10
3-Hexen-1-ol	Intense, grassy-green odor	Herbal	1.9	0.07±0.01	0.06±0.004	0.29±0.05	0.12±0.004	0.06±0.01	0.10±0.01	0.08±0.01	0.15±0.01	0.12±0.01	0.00±0.00	0.12±0.01	0.00±0.00	0.10±0.01	0.13±0.01	0.09±0.01	0.00±0.00	0.00±0.00
trans-2-Hexenol	Strong grassy-green odor, accompanied by fruit and vegetable odor	Herbal	4.2	0.00±0.00	0.00±0.00	0.00±0.00	0.00±0.00	0.00±0.00	0.03±0.002	0.00±0.00	0.00±0.00	0.00±0.00	0.03±0.001	0.00±0.00	0.00±0.00	0.00±0.00	0.00±0.00	0.00±0.00	0.00±0.00	0.00±0.00
1-Hexanol	Fruity odor and light grass odor	Fruity-sweet	0.2	0.00±0.00	0.00±0.00	1.69±0.35	1.06±0.11	0.99±0.25	0.00±0.00	0.00±0.00	1.27±0.11	0.00±0.00	0.97±0.05	0.00±0.00	1.14±0.40	0.00±0.00	0.57±0.12	1.77±0.08	1.25±0.09	0.00±0.00
1-Hexanol, 2-ethyl-	Sweet taste and light floral odor	Fruity-sweet	1.63	0.00±0.00	0.00±0.00	0.00±0.00	0.06±0.005	0.00±0.00	0.00±0.00	0.00±0.00	0.00±0.00	0.00±0.00	0.00±0.00	0.00±0.00	0.00±0.00	0.00±0.00	0.10±0.02	0.00±0.00	0.00±0.00	0.00±0.00

compounds was mainly derived by references [17, 43, 75].
om Van Gemert [76] and online database (http://www.vcf-online.nl/VcfHome.cfm)
old could not be obtained from references.

Table S6. Sequences of primers used in the qRT-PCR analysis.

Gene	Gene ID	Forward primer (5'-3')	Reverse primer (5'-3')
PlGADPH	-	TGTTCACCGACAAAGACAAGG	TAGCCAAGGGAGCAAGACAAT
PlDXS1	Isoform0006850	GCTGGATTTTGTTGCTTTCGT	TCGCCAACATCAGGTAAAGAA
PlDXS2	Isoform0020550	TATATTGGCCCTGTTGACGGT	TTTATCCACCTCTGCTTCTGC
PlDXS3	Isoform0025186	GGAGCATTTGACACCACTTAC	CCACCCTACTTCCCTCCTTT
PlDXR1	Isoform0016237	CGGGATGAATTTGTGAAGGGT	GGTGGCCAATGAAACGAGAAA
PlMCT1	Isoform0031172	CTGTGTCTCCATCCATCATCT	TTACTCTCTCCTTTCTCGCCA
PlCMK1	Isoform0026855	GGAACGGGCCAATAATTAGGA	GCAGAGCAAGTTCATGTGAGT
PlMDS1	Isoform0032368	GGAAATCTAGACGCCACCTT	TAAAAGTACAAACATCCGCCC
PlHDS1	Isoform0004050	GATGGCCACTGGAAATAAGA	ACCAGCAACCAACCAACTAA
PlHDR1	Isoform0030314	CAATTGGTGTTACATCTGGTG	CAACTCAGACATAAAAAGCCC
PlGPPS1	Isoform0022374	TGTGGCACTTGCTTCTTTGAG	AGCCAACATTGCAACTTCTGC
PlGPPS2	Isoform0024241	GGGTGGTCTAAAACTAATGTG	TCTGCTATAGCTCTCTCTCTT
PlGPPS3	Isoform0029797	ATTGATACACGATGACTTGCC	TCTTGCACAAAGTCTACCGAA
PlGPPS4	Isoform0031454	AAAATATTTTGATCAAGCTA	GAAAACAAAACCATAAACAT
PlTPS1	Isoform0014930	ATGAAGAGTTTGACCACGGAC	ATCGGCGGCATCCTGTTTGA
PlTPS2	Isoform0019028	ATGGCTACACGTCTGGTTGCT	GCATCGTCTTCACTTGTCTC
PlTPS3	Isoform0019388	ATGGCCATTTGCTCTGTGATT	CACCACTTTGGCAAGCTTCA
PlTPS4	Isoform0019653	ACGAGCCATCTCTCTCCATG	GGTCGCAATGGAGCTGTGAT
PlAADC1	Isoform0022490	ATTCTAGCGGTCGTGCATAC	GGTAGTAATTTGTGCCATCC
PlAADC2	Isoform0019696	CTACTCTTAAATAGACTATTA	GATAAAAAATAGTATACACGG
PlMAO1	Isoform0004723	CAAAGCTGTGATCAACGAGT	GTCCAATCTTGCAAAACGTC
PlCYP79-1	Isoform0021861	ATGGCCATCACTCTTGTATC	AGGGATGTTTCCAAGAATAG
PlPAR1	Isoform0031719	GACGAAGCTGTTGACATGTT	CCTCTTCCAGTAAATTTGCC
PlPAR2	Isoform0030865	AAGGTCTTTCATCCAAATAA	TTTGGATAAACATGCAAACA

PlPAR3	Isoform0030220	GCTGACTGCTAAAATGAACAC	ACAACTGGTTCTTTTTTCTTT
PlPAR4	Isoform0030470	GGCTATCTACCTGCCACTAC	GGAATACCAAGAAAACAATA
PlPAR5	Isoform0028371	TTACAGAAGTTGAAGTGTGG	GGAAATAATAACAAACAAAT
PlPAR6	Isoform0030732	TATTCTCTAAAACATTTGTA	ACGTGGTGAACATGACTTAA
PlPAR7	Isoform0029313	GGGAACTATGGTTGTGAAAA	ACATACATATCGGACAGACT
PlADH1	Isoform0028495	ATTTTTCCTATTTGATTTTC	AATCAAGTTCACAAGTCTTC
PlADH2	Isoform0028166	GTTGAGTTGTCTCTGAGCTA	AGAAGAAAATGAAGTTTAAA
PlADH3	Isoform0027181	GTACATTGAGAATAATCTGT	CCGGAAACAAATACGACTAA
PlADH4	Isoform0028765	TTGAGTACATGATTAAGGGG	GTGAACCTCATCAAAATGTA
PlADH5	Isoform0028314	GGAGGTCATCCGAAAAATTA	TAACTTGACCTTGGGTAGCC
PlADH6	Isoform0026717	TTCTTAGGGCTGGGGAATAT	CTAGGAAAAAAAGATCAAAT
PlADH7	Isoform0025289	GCTGTGGTACACAAGTGTTG	GAAAAAAGTTGCCCAGTTTT
PlADH8	Isoform0029306	ATAATAATATCGGTAAGACG	GTCGATATCTAACCAGGTAT
PlADH9	Isoform0028507	TCACCATCATCAATGTTTTT	GAAATACCATCCAAAAGTAT
